# Deep reinforcement learning framework for joint optimization of multi-RAT UAV location and user association in heterogeneous networks

**DOI:** 10.1038/s41598-025-22610-1

**Published:** 2025-11-07

**Authors:** Mohamed G. Anany, Mahmoud M. Elmesalawy, Ahmed M. Abd El-Haleem, Ibrahim I. Ibrahim

**Affiliations:** 1 Department of Communications and Electronics, Canadian International College, CIC, Cairo, 11865 Egypt; 2 Department of Electronics, Communications and Computer, Faculty of Engineering, Helwan University, Cairo, 11722 Egypt

**Keywords:** Engineering, Electrical and electronic engineering

## Abstract

The explosive growth of multimedia and Internet of Thing (IoT) devices has led to a huge increase in data traffic requirements with a reduced power consumption demands in 6G communications. In this work, a ground Multiple Radio Access Technology (Multi-RAT) Heterogeneous Network (HetNet) is considered, which is assisted by multiple UAVs, each carrying Multi-RAT base stations (i.e., LTE and Wi-Fi base stations), to utilize the unlicensed spectrum, and provide an on-demand assistance, more capacity, and coverage for diverse ground devices. A Satisfaction to Energy Ratio (SER) is introduced, which is a ratio between the users’ satisfaction according to their requirements, and the UAVs’ energy consumption. An iterative framework is proposed to maximize the SER by optimizing the UAVs 3D location and the users association. The proposed framework uses a modified K-means algorithm for initialization, Deep Reinforcement Learning (DRL) to optimize the 3D location of UAVs, and regret learning to optimize the user association. Extensive simulations show an improvement percentage that reaches 13%, 25%, 67%, 71%, 28%, 45% in satisfaction index, downlink data rate, uplink power consumption, outage probability, Jain’s fairness index, and framework iterations, respectively. In addition, a comparison between different DRL algorithms, observation scenarios, and training approaches is presented to select the best combination of them in the proposed framework.

## Introduction

The next generation of wireless communication networks, 6G, is expected to support a wide range of applications with diverse requirements, such as ultra-high-speed data transmission, low-latency communication, and massive connectivity. To meet these requirements, Multi-Radio Access Technology (Multi-RAT) Heterogeneous Networks (HetNets) have been proposed as a promising solution. HetNets represents base stations with different area coverage (i.e., Macro, Micro, and Femto base stations), while Multi-RATs combine different wireless access technologies, such as cellular, Wi-Fi, to provide seamless and ubiquitous connectivity to users. However, the deployment and operation of HetNets pose significant challenges, particularly in terms of optimizing the network coverage, capacity, and Quality of Service (QoS).

Unmanned aerial vehicles (UAVs) have emerged as a promising technology for providing wireless communication services in areas with limited or no network coverage^1^. Due to their flexibility, mobility, UAVs can be deployed as aerial base stations to enhance the coverage and capacity of existing wireless networks, especially in Multi-RAT HetNets^2^. Additionally, the high altitude of UAV-carried flying BSs enables Line of Sight (LoS) to ground devices (GDs), making them easily distinguishable at different altitudes and elevation angles^3,4^.

In this paper, a novel approach for optimizing the location and GD association of multiple UAVs in Multi-RAT HetNets is proposed. Our approach leverages deep reinforcement learning algorithms to dynamically adjust the UAVs’ locations and GD associations based on the network conditions and GD demands. The performance of our approach is evaluated through extensive simulations that demonstrate its effectiveness in improving the different network metrics, and GD experience. By addressing the challenges of deploying multiple UAVs in a ground Multi-RAT HetNet environment, our approach can pave the way for the efficient and effective deployment of UAVs in future wireless communication networks. To enhance the readability of the paper, Table 1 provides a list of the common abbreviations.

**Table 1:** List of common abbreviations.

Abbreviation	Full name	Abbreviation	Full name
6G	Next Generation of Wireless Communication Networks	MDP	Markov Decision Process
AC	Actor-Critic	Multi-RAT	Multi-Radio Access Technology
CDF	Cumulative Distribution Function	NOMA	Non-Orthogonal Multiple Access
C-NOMA	Clustered-Non-Orthogonal Multiple Access Technique	QoS	Quality of Service
CQI	Channel Quality Indicator	RTS	Request-To-Send
DDPG	Deep Deterministic Policy Gradient	SER	Satisfaction-to-Energy Ratio
DFL	Distillation Federated Learning	SINR	Signal to Interference plus Noise Ratio
DRL	Deep Reinforcement Learning	SNR	Signal to Noise Ratio
FBSs	Femto Base Stations	TDD	Time Division Duplexing
FL	Federated Learning	UBSs	UAV Base Stations
HAPS	High Altitude Platform Station	UFBSs	UAV Femto Base Stations
HetNets	Heterogeneous Networks	UWAPs	UAV Wi-Fi Access Points
MBS	Macro Base Station	VoWiFi	Voice over Wi-Fi

## Literature review

This section provides classified related works along with the concluded research gap, followed by the contribution of this work.

### Related work

In this subsection, recent works of UAV base stations that use the licensed band will be presented. Followed by works with UAV base stations that exploit the unlicensed band. After that, works that use Deep Reinforcement Learning (DRL) algorithms will be presented. In the end, all the related work will be summarized to describe the research gap.

#### Licensed band UAV base stations

In^5^, the authors considered a Clustered-Non-Orthogonal Multiple Access Technique (C-NOMA) heterogeneous air-to-ground integrated network, including one high altitude platform Station (HAPS) for backhaul, and multiple UAVs as base stations for access. The aim was to maximize energy efficiency by optimizing the joint UAV trajectory plane and resource allocation problem. After decoupling the problem into two subproblems, the optimal channel and power strategy are obtained according to the Lagrange dual decomposition method, while a near-optimal UAV trajectory and flight speed are obtained based on successive convex approximation methods. In^6^ , the authors proposed an outer approximation algorithm to optimize the phone user’s admission, cell association, throughput, and energy efficiency while ensuring users fair association with cells, and their minimum rate requirement, in UAV-assisted HetNets. In^7^ , the authors proposed an outer approximation algorithm to maximize the network data rate, subject to the constraints of power and QoS, by optimizing the resource allocation of a UAV-assisted HetNet environment.

#### Unlicensed band UAV base stations

Paper^8^ proposed heuristic algorithms, including K-means and genetic algorithms, to find the optimal solutions of the minimum number of UAVs that can provide Voice over Wi-Fi (VoWiFi) services to GDs, subject to coverage, call blocking probability, and QoS constraints. The authors in^9^ considered a two-layered architecture, where access UAVs provide Wi-Fi access to GDs, while distribution UAVs act as Wi-Fi-to-5G relays, to forward packets to the core network. They used a metaheuristic Particle Swarm Optimization (PSO) algorithm to find the minimum number of UAVs, their type, and their locations, constrained to coverage and minimum voice speech quality for VoWiFi services to GDs.

In^10^ , the authors proposed a power allocation and time allocation scheme to maximize the overall UAV-assisted Internet of Vehicles (IoVs) system capacity, considering Road Side Units (RSUs) that can properly occupy the unlicensed band to mitigate the interference between the UAVs and the RSUs.

In^11^ , the authors investigated how to deploy UAVs mounted Wi-Fi AP efficiently for maximizing the sum throughput and service time of mobile users. A DRL-based chunk selection algorithm was proposed to select the optimal subset of chunks in a region as the search space for UAVs, while an energy-aware DRL chunk search algorithm was proposed to plan the path for the UAVs to cover the selected chunks.

The authors in^12^ exploited the New Radio Unlicensed (NRU) technology proposed in 3GPP Release 16^13^ in a UAV HetNet environment. They developed a mathematical framework that characterizes the medium access and coverage probability of the aerial and terrestrial base stations utilizing the NRU and the licensed spectrum. Also, in^14^ , the user to UAV uplink sum rate was maximized by jointly optimizing the power control and subchannel allocation over licensed and unlicensed channels in a terrestrial cellular, Wi-Fi, and UAV base stations environment.

#### Deep reinforcement learning

The authors in^15^ adopted an Actor-Critic (AC) algorithm that optimizes the UAV’s trajectory to enhance the security of the user’s information and their QoS, based on their movement and density, considering a heterogeneous UAV-assisted network. In addition, Federated Learning (FL) and Distillation Federated Learning (DFL) algorithms are combined with the AC algorithm to improve the users’ QoS and increase the security, accuracy, and speed of learning.

Paper^16^ proposed a combination of DRL and fixed-point iteration techniques for optimizing the UAVs’ locations and the channel allocation strategies, in order to maximize the user’s fairness and load balance of the aerial base stations in a heterogeneous HAPS-UAV network. The work in^17^ considered a single UAV-assisted heterogeneous network in a disaster area, serving ground cellular users, and sensor users. For users outside UAV’s coverage, a multi-hop relay transmission is adopted by selecting the energy-effective relays. For users with different requirements inside the UAV’s coverage, a DRL approach is adopted to reduce energy consumption and improve QoS satisfaction by adjusting the power level and allocated sub-bands considering a NOMA transmission.

In^18^, the authors considered the deployment of UAV base stations to provide on-demand communications to ground users in emergency scenarios. A DRL approach was proposed to maximize a weighted sum of the backhaul link rate, the users’ throughput, and the users’ drop rate, by optimizing the UAVs’ 3-D locations. The work in^19^ investigated the use of the Double Deep Q-Network (DDQN) technique, to optimize the UAV height, and the resource allocation, with the aim of maximizing the energy efficiency and the total network throughput in UAV-assisted terrestrial networks.

To summarize, the works^5–7^ considered investigating environments where UAVs act as base stations that work on the licensed spectrum. Papers^8–14^ considered exploiting the unlicensed band in UAV communications. In addition, the studies^15–19^ considered using continuous and discrete action space DRL algorithms to optimize the UAV’s location or trajectory in various environments. Notably, most of the aforementioned works considered objectives like sum rate, uplink power consumption, energy efficiency, and QoS.

From the aforementioned works, a research gap can be noticed, where none of them considered the deployment of both LTE and Wi-Fi base stations on each UAV, exploiting the unlicensed band, and increasing the system capacity, with almost the same operating cost, which can be called on demand whenever there is a sudden surge in traffic, or in case of disasters. Besides, none of them considered the comparison between discrete and continuous action space DRL techniques, their observations (i.e., complete or incomplete information), and their training approaches (i.e., centralized and decentralized), in such deployment scenario where multiple UAVs are considered. Especially, when system models use more practical downlink data rate calculations than using the theoretical Shannons equations, which affects the environment structure, and accordingly the better technique to be used.

### Contribution

In this work we study and investigate the idea of deploying Multi-RAT base stations on each UAV, considering a Multi-UAVs assisted Multi-RAT HetNet. Terrestrial base stations are composed of Macro Base stations (MBSs), Femto base stations (FBSs), and Wi-Fi access points (WAPs), while multiple UAVs act as aerial base stations, each carrying an LTE and Wi-Fi base station. Different ground devices are considered, that have diverse requirements.

Although deploying Multi-RAT base stations on each UAV is cost-effective, and offers a higher degree of freedom compared to fixed-ground base stations, it is also challenging to find optimum locations for these UAVs that satisfy both LTE and Wi-Fi GDs at the same time^3^. Besides, it has always been challenging to find the optimum GD association considering the movement of UAVs and the diverse GD requirements. In particular, the contribution of this work is summarized below.

1. To the best of our knowledge, the idea of Multi-RAT base stations deployment on each UAV, in a Multi-UAV acting as aerial base stations, assisting a Multi-RAT HetNet terrestrial environment has not been investigated. Therefore, in this work, the effectiveness of deploying LTE and Wi-Fi base stations on each UAV, considering a Multi-RAT HetNet ground environment is evaluated and investigated, considering different wireless network metrics.
2. Since different GDs with diverse requirements are associated with each UAV, a Satisfaction-to-Energy-Ratio (SER) is proposed to calculate the associated GDs’ satisfaction with respect to a UAV’s consumed energy. To quantify the GDs’ satisfaction, a satisfaction index is introduced to measure the GDs’ satisfaction according to their requirements, in terms of achievable downlink data rate, uplink power consumption, downlink Signal to Interference Noise Ratio (SINR), and uplink Signal to Noise Ratio (SNR), while taking into consideration the dissimilar access techniques (i.e., LTE and Wi-Fi).
3. A framework that combines K-means, multi-agent DRL, and regret learning algorithms, is developed to find the initial GDs association and UAVs 3D locations, the optimized UAVs 3D location, and the optimized GDs association, respectively. The objective is to maximize the GDs’ satisfaction and minimize the UAVs’ energy consumption (i.e., maximize the total SER).
4. Evaluating the adoption of discrete actions deep reinforcement learning algorithm, by comparing it with a continuous one, under different observation scenarios and learning approaches, considering multiple UAVs, and a more practical system model for LTE and Wi-Fi technologies than the well-known Shanons theory.

The rest of the paper is organized as follows: (System architecture and problem formulation) section describes the system architecture and the problem formulation. Followed by modeling the downlink data rate, the uplink power consumption, and the UAV’s energy consumption in (System model). After that, the developed framework to solve the optimization problem is presented in (DRL-regret learning framework). Finally, the performance of the proposed work is evaluated in (Performance evaluation and discussion).

**Fig. 1:**
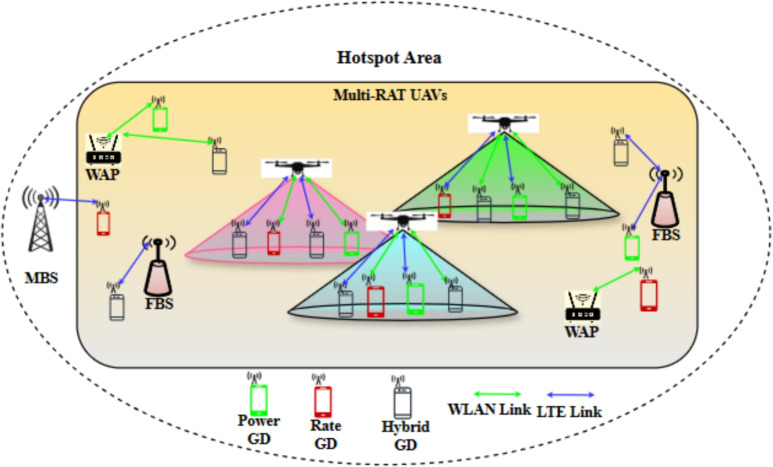
System architecture of Multi-RAT UAVs assisted Multi-RAT HetNet serving diverse GDs.

**Table 2:** Commonly used notations and variables.

Notation	Description	Notation	Description
$\mathcalligra{\scriptstyle K} \hspace{000.1cm}, \mathcalligra{\scriptstyle L} \hspace{000.1cm}, \mathcalligra{\scriptstyle W} \hspace{000.1cm},\mathcalligra{\scriptstyle U} \hspace{000.1cm}, \mathcalligra{\scriptstyle G} \hspace{000.1cm}$	The set of BSs, FBSs, WAPs, UBS, and GDs.	*X*, *Y*, *H*	The UAVs’ X-axis, Y-axis, and altitude.
*K*, *L*, *U*, *N*	The number of BSs, LTE-BSs, UBSs, and GDs.	$P^w,P_{i,k}^u$	Wi-Fi card, and average uplink power consumption.
$N^K, N_k^L,$ $N_k^W$	The number of associated GDs with MBS, FBS, and WAP *k*.	$R_i^u,\Gamma ^u$	GD’s uplink average traffic generation rate, and target SNR.
$P_k\left( V_k\right) ,E_k$	UAV’s power consumption and energy consumption.	$\mathcalligra{\scriptstyle S} \hspace{000.1cm},\mathbb {A},\mathbb {O}$	Set of the state, joint action, and joint observation spaces.
*s*(*t*), *a*(*t*), *r*(*t*)	The state, action, and reward at time *t*.	$\gamma ,\pi ,\pi ^*$	Discount factor, UAV’s policy, and optimal policy.
$\theta , \theta ^-$	$\mathcalligra{\scriptstyle Q} \hspace{000.1cm}$-network, and target network weights.	$\mathcalligra{\scriptstyle Q} \hspace{000.1cm}^\pi (s,a)$	The UAV’s state-action value function.
$M^{ep}$	The number of episodes of the Q-learning algorithm.	$\hspace{000.2cm} \mathcalligra{\scriptstyle J} \hspace{000.2cm}$	The regret-matching game.
$R_{ik}^d,R_{ik}^{WPHY},S_i$	The average downlink data rate, downlink WLAN physical data rate, GD’s satisfaction.	$D_i^t\left( m_i,m_i^\prime \right)$	The payoff for GD *i* if it had played action $m_i$ instead of $m_i^\prime$.
$T_{SC}^{LTE}$	The duration of an LTE subframe.	$\psi _i^{t+1}\left( m_i\right)$	The probability distribution of GD *i* choosing an action at time *t*.
$C_{i,k}^{LMCS}, C_{i,k}^{WMCS}$	The coding rate of LTE BSs and WAPs.	${\bar{z}}_t$	The empirical distribution of joint actions $\Sigma$ of all GDs until *t*.
*A*	Association matrix between GDs and base stations.	$\Sigma ^*$	Optimal joint strategies.

## System architecture and problem formulation

In this section, a detailed description of the proposed system architecture is presented. Followed by formulating the optimization problem.

### System description

In this paper, multiple Multi-RAT UAVs are exploited to assist a ground Multi-RAT HetNet are considered, such that each UAV carries an FBS and a WAP, to offer diverse connectivity (i.e., LTE and Wi-Fi technologies) and maximum capacity (i.e., by utilizing the unlicensed spectrum band) for GDs. The ground Multi-RAT HetNet is comprised of multiple FBSs and WAPs overlaid by a Macro Base Station (MBS), as shown in Fig. 1.

To ease the readability, Table 2 shows the list of notations. In general, $\mathcalligra{\scriptstyle K} \hspace{000.1cm}=\{1,2,\ldots ,k,\ldots ,K\}$ denotes the set of all available base stations in the system, with cardinality *K*, such that $k=1$ represents the MBS, while other base stations can be represented by $k=2,\ \ldots ,K$. The aerial and ground FBSs can be denoted by $\mathcalligra{\scriptstyle L} \hspace{000.1cm}=\{2,\ldots ,L\}$, with cardinality $L-1$, such that $\mathcalligra{\scriptstyle L} \hspace{000.1cm}\subset \mathcalligra{\scriptstyle K} \hspace{000.1cm}$. Also, the set $\mathcalligra{\scriptstyle W} \hspace{000.1cm}=\{L+1,L+2,\ldots ,K\}$ denotes the aerial and ground WAPs, with cardinality $K-L$, where $\mathcalligra{\scriptstyle W} \hspace{000.1cm}\subset \mathcalligra{\scriptstyle K} \hspace{000.1cm}$. Moreover, the UAVs Base Stations (UBSs) are denoted by the set $\mathcalligra{\scriptstyle U} \hspace{000.1cm}=\{L-U+1,\ L-U+2,\ldots ,L+U\}$, with cardinality 2*U*, where *U* is the number of UAVs, such that $\mathcalligra{\scriptstyle U} \hspace{000.1cm}\subset \mathcalligra{\scriptstyle K} \hspace{000.1cm}$. The UAVs Femto Base Stations (UFBSs) are those from $L-U+1$ to *L*, while those from $L+1$ to $L+U+1$ are the UAVs Wi-Fi Access Points (UWAPs). A set $\mathfrak {U}=\{1,2,\ldots ,u,\ldots ,U\}$ denotes the set of UAVs in general, such that UAV *u* carries FBS $L-U+u$ and WBS $L+u$.

Without loss of generality, Time Division Duplexing (TDD) is considered as the duplexing mode in the LTE system, and the distributed coordination function (DCF) mechanism with Request-To-Send (RTS)/Clear-To-Send (CTS) handshaking is considered as the access mechanism in Wi-Fi. Spare and fully charged UAVs are considered to be ready to replace the working UAVs when their energy reaches a predefined critical level. The UAVs are assumed to efficiently carry the Multi-RAT base stations. In addition, a robust, secure and reliable common control channel is assumed for communication and coordination between UAVs and a common control station, that has information about all the UAVs locations and their trajectories. The common control station is critical since it supports UAVs with traffic coordination and send alerts to avoid collision, whenever the probability of collision between the UAVs increases.

Meanwhile, the set of GDs is denoted by $\mathcalligra{\scriptstyle G} \hspace{000.1cm}=\{1,2,\ldots ,i\ldots ,N\}$, with cardinality *N*, where *N* is the total number of GDs. And, $N^K$, $N_k^L$, and $N_k^W$ denote the number of associated GDs with the MBS, an FBS, and a WAP, respectively. Due to the diversity of the GDs in reality, different requirements for GDs are considered. For example, sensors, IoT devices, and mobile phones with low batteries require low power consumption, while H2H, and mobile phones with data-hungry applications may pay more attention to high data rates, rather than power consumption. The GDs are assumed to be capable of connecting via both, LTE, or Wi-Fi.

### Problem formulation

In this work, we focus on finding the optimum UAVs’ 3D locations jointly with the optimum GDs’ association, to maximize the total SER, which is a ratio between the sum of the GDs’ satisfaction index, and the sum of the UAVs’ energy consumption. The UAVs’ X-axis, Y-axis, and altitude location sets are denoted by ${\mathbf{X}}$, ${\mathbf{Y}}$, and,${\mathbf{H}}$, respectively. Also, the ground devices’ association matrix is denoted by *A*, such that *A* is an $N\times \ M$ matrix. The problem can then be defined mathematically as follows:

1 $$\begin{aligned}&\mathbf{OPT}:\max _{ A,\mathbf{X},\mathbf{Y},\mathbf{H}}{\frac{\sum _{i,k}{A_{i,k}S_{i,k}}}{\sum _{k\in \mathcalligra{\scriptstyle U} \hspace{000.1cm}}\ E_k}} \end{aligned}$$

2 $$\begin{aligned} s.t.,\nonumber \\&\sum _{k\in \mathcalligra{\scriptstyle K} \hspace{000.1cm}}\ A_{i,k}=1,\ \ \forall k\in \mathcalligra{\scriptstyle K} \hspace{000.1cm},i\in \mathcalligra{\scriptstyle G} \hspace{000.1cm}, \end{aligned}$$

3 $$\begin{aligned}&A_{i,k}=\left\{ 0,1\right\} ,\ \ \forall k\in \mathcalligra{\scriptstyle K} \hspace{000.1cm},i\in \mathcalligra{\scriptstyle G} \hspace{000.1cm},\end{aligned}$$

4 $$\begin{aligned}&\textrm{SNR}_{ik}>\textrm{SINR}_k^{thr},\ \ \ \forall k\in \mathcalligra{\scriptstyle K} \hspace{000.1cm},i\in \mathcalligra{\scriptstyle G} \hspace{000.1cm},\end{aligned}$$

5 $$\begin{aligned}&P_{i,k}^u\le \ P_i^{u,max},\ \forall k\in \mathcalligra{\scriptstyle K} \hspace{000.1cm},i\in \mathcalligra{\scriptstyle G} \hspace{000.1cm}, \end{aligned}$$

where, $S_{i,k}$ is the satisfaction index of GD *i*, and it can be calculated by $S_i=\zeta _i^R\left( 1-e^{-\frac{R_{i,k}^d}{\kappa (R^{ref})}}\right) -(1-\ \zeta _i^R)\frac{P_{im}^u}{P_{i,max}^u}$, where $\zeta _i^R=[0,1]$ represents the weight value of the downlink data rate $R_{i,k}^d$ for GD *i* connected to base station *k*, considering a reference downlink data rate $R^{ref}$ the operator would like GDs’ to achieve, and $(1-\ \zeta _i^R)$ is the weight toward its uplink power consumption. This implies that the GD’s satisfaction relies on its requirements and considerations, which differ from one GD to another and may change with time. $E_k$ is the energy consumption of UAV $k\in \mathcalligra{\scriptstyle U} \hspace{000.1cm}$. Constraints 2 and 3 ensure that a GD *i* must be associated with only one base station, where the association index $A_{ik}= 0$ indicates that GD *i* is unassociated with BS *k*, while $A_{ik}=1$ indicates that GD *i* is associated with BS *k*. In addition, constraint 4 ensures that GDs will be provided with at least the minimum SINR value to maintain their connection. In this regard, this constraint limits the UAVs movement, to ensure that all GDs associated with UAVs do not lose their connection. And, to ensure that GDs associated with ground BSs will not lose their connection due to the change in interference caused by the UAVs’ movements. Moreover, constraint 5 ensures that the GD’s uplink transmitted power is below the maximum allowed. Notably, the optimization problem OPT is a non-convex integer programming optimization problem, which is difficult to be optimally solved in general. Besides, the optimal UAV location problem is proved to be an NP-hard problem^20^.

**Fig. 2:**
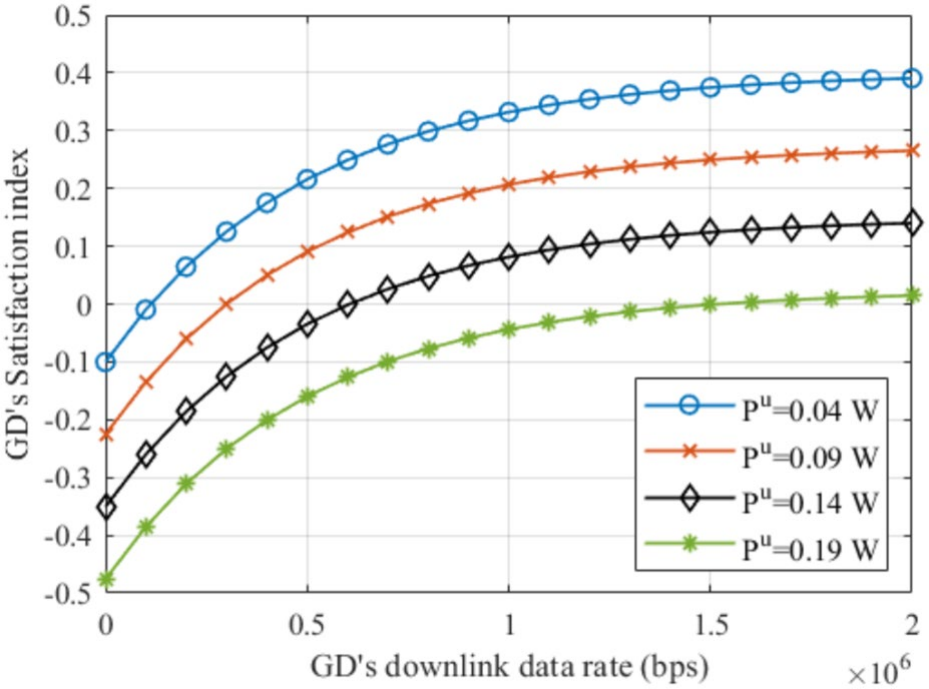
Satisfaction index with the downlink data rate at different uplink power consumption.

Figure 2 shows how the satisfaction index increases with the increase in the downlink data rate at different uplink power consumption at $\zeta _i^R=0.5$ and $R^{ref}=1Mbps$. It can be noticed that before 1Mbps the satisfaction gains by increasing the data rate are greater compared to after achieving $R^{ref}$, this is due to the benefits gained by the GD when it is striving for increasing the data rate. Also, it can be noticed that the gaps between the curves are equal, which indicates the linearity of decreasing the uplink power consumption.

## System model

In this section, a detailed modeling of the proposed system architecture is presented.

### Data rate modelling

In this work, radio channels are allocated equally to the GDs associated with any LTE-BS (i.e., MBS, FBSs, and FUBSs). In addition, for each LTE-BS, the transmission power is allocated equally to the available radio channels, and a frequency reuse factor of 1 is considered. Moreover, the path loss models and the channel gains are calculated as^21^. Therefore, considering interference, the downlink average SINR at GD $i\in \mathcalligra{\scriptstyle G} \hspace{000.1cm}$ served by LTE-BS $k\in \mathcalligra{\scriptstyle L} \hspace{000.2cm}$ can be calculated as in^22,23^, and it can be denoted by $\textrm{SINR}_{ik}$.

For modeling the downlink data rate for GDs, and since different technologies are considered, we adopt the same modeling as in^22^, which considers various RAT parameters rather than using Shannon’s capacity formula for the different RATs. Thus, the average downlink data rate achieved by GD *i* from any LTE-BS $k\in \mathcalligra{\scriptstyle K} \hspace{000.2cm}\backslash \mathcalligra{\scriptstyle W} \hspace{000.1cm}$ can be given in bits per seconds by^22^:

6 $$\begin{aligned} R_{i,k}^d=\frac{N_k^{SC}.N_{sym}.b_{i,k}^{LMCS}.C_{i,k}^{LMCS}}{N_k^L.T_{SC}^{LTE}},\ \ \ \ \forall k\in \mathcalligra{\scriptstyle K} \hspace{000.2cm}\backslash \mathcalligra{\scriptstyle W} \hspace{000.1cm}, \end{aligned}$$

where $N_k^{SC}$ is the number of subcarriers, $N_{sym}$ is the number of OFDM symbols in one subframe, $T_{SC}^{LTE}$ is the duration of one subframe and is typically equal to 1ms, $b_{i,k}^{LMCS}$ is the number of bits in one symbol, and $C_{i,k}^{LMCS}$ is the coding rate, both obtained from LTE Modulation and Coding Scheme (MCS). In particular, $b_{i,k}^{LMCS}$ and $C_{i,k}^{LMCS}$ are mapped to the Channel Quality Indicator (CQI) index which can be determined from the SINR values, representing the radio link quality.

On the other hand, for calculating the WLAN downlink data rate, DCF channel contention is considered, and WAPs are considered to grant different non-overlapping channels, hence, interference from other WAPs is neglected as in^24^. Therefore, the downlink average SNR for GD *i* served by WAP $k=\mathcalligra{\scriptstyle W} \hspace{000.1cm}$ can be calculated as in follows^24^, and it can be denoted by $\textrm{SNR}_{ik}$. The physical downlink data rate for GD *i* served by WAP $k\in \mathcalligra{\scriptstyle W} \hspace{000.1cm}$ in bits per seconds can then be calculated as follows:

7 $$\begin{aligned} R_{i,k}^{WPHY}=\ \frac{{N_{i,k}^{sp}.\ N}_{SC}.\ b_{i,k}^{WMCS}.C_{i,k}^{WMCS}\ }{T_{sym}},\ \forall k\in \mathcalligra{\scriptstyle W} \hspace{000.1cm} \end{aligned}$$

where $N_{i,k}^{sp}$ is the number of available spatial streams, $N_{SC}$ is the total number of data subcarriers, $b_{i,k}^{WMCS}$ is the number of bits in one symbol, $C_{i,k}^{WMCS}$ is the coding rate, and $T_{sym}$ is the OFDM symbol duration. Similarly, $b_{i,k}^{WMCS}$, and $C_{i,k}^{WMCS}$ are mapped to the CQI index which can be determined from the SNR values according to Wi-Fi MCS.

In addition, the downlink data rate achieved by GD *i* from WAP $k\in \mathcalligra{\scriptstyle W} \hspace{000.1cm}$ can be formulated in bits per seconds as in^22^, where the WLAN MAC layer effect on the downlink data rate is considered as follows:

8 $$\begin{aligned} R_{i,k}^d=\frac{E(N_k^W)}{\left( T+\left( \frac{E(N_k^W)}{R_{i,k}^{WPHY}}\right) \right) }\ \ \ \ \forall k\in \mathcalligra{\scriptstyle W} \hspace{000.1cm} \end{aligned}$$

where $E(N_k^W)=\tau {(1-\tau )}^{N_m^W}D$ is the average per-GD data transferred in a time slot. $\tau$, and *D* are the channel contention probability, and the maximum allowable packet size, respectively. $E(N_k^W)$ represents the probability of successful transmission occurring in a time slot, multiplied by the probability that GD *i* is transmitting, multiplied by *D*. The denominator in 8 represents the average length of a time slot, where the term $\frac{E(N_k^W)}{R_k^{WPHY}}$ is written in terms $R_{i,k}^{WPHY}$ represents the duration of successful data transmission. Also, *T* can be calculated in seconds as in^22,23^ by:

9 $$\begin{aligned} T={(1-\tau )}^{N_k^W+1}e+\left( 1-\left( 1-\tau \right) ^{N_k^W+1}\right) \left( T_{RTS}+T_{DIFS}\right) + \left( N_k^W+1\right) \tau \left( 1-\tau \right) ^{N_k^W}(T_{CTS}+T_{ACK}+3T_{SIFS}) \end{aligned}$$

where *e* is the duration of an empty slot time; $T_{RTS}$, $T_{DIFS}$, $T_{CTS}$, $T_{ACK}$, and $T_{SIFS}$ are the durations of RTS short frame, DCF Interframe Space, CTS short frame, Acknowledgment short frame, and Short Interframe Space, respectively.

### Uplink transmitted power

Depending on the GD’s association (i.e., connected to an LTE-BS, or a WAP), the uplink transmit power of GD *i* can be deduced. When GD *i* is associated with a WAP $k\in \mathcalligra{\scriptstyle W} \hspace{000.1cm}$, the Wi-Fi cards transmit a constant uplink power $P^w$. However, due to collision, the uplink power transmitted differs from one GD to another. Considering a packet collision probability $p(N^c)$, a packet will be averagely transmitted $E\left( p\right) =\frac{1-{p(N^c)}^{\mu +1}}{1-p(N^c)}$ times until it is successively received by WAP^22^. Since the probability of dropping a frame after m retransmissions is negligible, ${p(N^c)}^{\mu +1}$ could be ignored. Hence, the uplink transmitted power consumed by GD *i* connected to WAP $k\in \mathcalligra{\scriptstyle W} \hspace{000.1cm}$ in watts can be calculated as follows^22^:

10 $$\begin{aligned} P_{i,k}^u=\frac{R_i^uP^w}{R_{i,k}^{WPHY}(1-p(N^W))},\ \ \ \forall k\in \mathcalligra{\scriptstyle W} \hspace{000.1cm} \end{aligned}$$

where $R_i^u$ is the uplink average traffic generation rate for GD *i*, and it can be calculated by $R_i^u=\frac{\lambda _iD}{T}$ in bits per seconds, where $\lambda _i$,*T* are the packets generation rate (packets/slot) for GD *i*, and the backoff average duration time (seconds/slot), respectively. And, $\frac{R_i^u}{R_{i,k}^{WPHY}(1-p(N^c))}$ is the ratio of time GD *i* is in transmission state.

On the other hand, since open loop power control is used by LTE-BSs, we consider a target SNR $\Gamma ^u$ at the LTE-BS for associated GDs. In the uplink, we assume resources are allocated to minimize the uplink interference, thus, interference can be neglected. Therefore, the SNR can be represented in dBs as follows^22^:

11 $$\begin{aligned} \Gamma ^u=\frac{P_i^ug_{i,k}}{B_{i,k}\sigma ^2},\ \ \ \forall k\in \mathcalligra{\scriptstyle K} \hspace{000.2cm}{\backslash } \mathcalligra{\scriptstyle W} \hspace{000.1cm} \end{aligned}$$

where $P_i^u$ is the uplink transmitted power for GD *i*; $B_{i,k}=N_k^{SC}.\frac{f_{spac}^{LTE}}{{N_k^L}}$ is the allocated bandwidth to GD *i* by LTE-BS $k\in \mathcalligra{\scriptstyle K} \hspace{000.2cm}{\backslash }\mathcalligra{\scriptstyle W} \hspace{000.1cm}$, to meet its uplink rate requirement $R_{i,k}^u$, and it can be represented by the number of allocated subcarriers multiplied by the frequency spacing $f_{spac}^{LTE}$ for each subcarrier. Recall that, the uplink rate requirement can be calculated in bits per seconds similarly to 6 by:

12 $$\begin{aligned} R_{i,k}^u=\frac{B_{i,k}\ .\ \ N_{sym}\ .\ \ b_{i,k}^{LMCS}.\ \ {C}_{i,k}^{LMCS}}{f_{spac}^{LTE}\ .\ \ T_{SC}^{LTE}},\ \ \forall k\in \mathcalligra{\scriptstyle K} \hspace{000.2cm}{\backslash }\mathcalligra{\scriptstyle W} \hspace{000.1cm} \end{aligned}$$

By substituting $B_{i,k}$, the uplink transmitted power by GD *i* to LTE-BS $k\in \mathcalligra{\scriptstyle K} \hspace{000.2cm}{\backslash }\mathcalligra{\scriptstyle W} \hspace{000.1cm}$ in watts can be formulated as follows:

13 $$\begin{aligned} P_{i,k}^u=\frac{\Gamma ^u\sigma ^2}{g_{i,k}}.\frac{R_{i,k}^u\ .{\ f}_{spac}^{LTE}\ .\ \ T_{SUB}^{LTE}}{N_{SC}\ .{\ B}_i^{LMCS}.\ \ {C}_i^{LMCS}},\ \ \forall k\in \mathcalligra{\scriptstyle K} \hspace{000.2cm}{\backslash }\mathcalligra{\scriptstyle W} \hspace{000.1cm} \end{aligned}$$

### UAV energy consumption model

Generally, the energy consumption of a rotary wing UAV can be divided into communication-related energy and propulsion energy. In this work, the communication-related power is neglected, since it has a notably smaller effect on the total UAV power consumption compared to the propulsion power^25^. Therefore, the UAV power consumption model based on the propulsion power can be calculated in watts as follows^25^:

14 $$\begin{aligned} P_k\left( V_k\right) =\underbrace{P_0\left( 1+\frac{3V_k^2}{U_{ip}^2}\right) }_{blade\ profile\ power}+\underbrace{P_{in}\left( \sqrt{1+\frac{V_k^4}{v_0^2}}-\frac{V_k^2}{2v_0^2}\right) ^\frac{1}{2}}_{induced\ power}- \underbrace{\frac{1}{2}r_0\varrho sa V_k^3}_{parasite\ power},\ \ \ \forall k\in \mathfrak {U} \end{aligned}$$

where $V_k$ is the velocity of UAV *k*, $P_0$ and $P_{in}$ are the blade profile power and induced power constants in the hovering state, respectively, $U_{tip}$ represents the rotor blade tip speed, $v_0$ denotes the mean rotor induced velocity in hovering state, $r_0$ is the fuselage drag ratio, $\varrho$ is the air density, *s* is the rotor solidity, and a denotes the rotor disc area. It can be noticed that the UAV propulsion power consumption combines three main power components, the blade profile power, induced power, and the parasite power. These powers act differently with the increase of $V_k$, where the blade profile and parasite powers increase quadratically and cubically, respectively, while the induced power decreases with the increase of $V_k$.

As concluded in^25^, two UAV speeds are of high interest, the maximum endurance speed $V_{me}$, which is the optimal UAV speed that minimizes power consumption, and the maximum range speed $V_{mr}$, which is the optimal UAV speed that maximizes the total travelling distance under any onboard energy. Hence, the energy consumption of the rotary wing UAV can be then calculated in joules by:

15 $$\begin{aligned} E_k=\frac{P_k\left( V_k\right) .d_k^{uav}}{V_k},\forall k\in \mathfrak {U} \end{aligned}$$

where $d_k^{uav}$ is the distance traveled by UAV *k*. Thus, $V_{mr}$ is used when traveling to the targeted points determined by the proposed algorithms.

## DRL-regret learning framework

**Figure 3:**
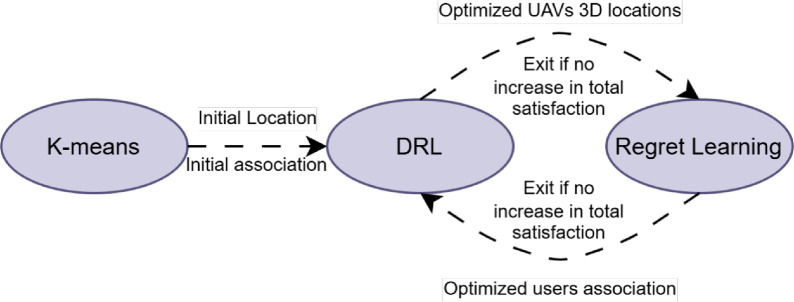
DRL-regret learning framework.

Since the optimization problem OPT is hard to solve jointly due to the interdependency between the UAVs’ locations and the GDs’ association, an iterative framework based on DRL and regret learning techniques is proposed, to optimize the UAVs’ locations and GDs’ association, respectively, as shown in Fig. 3. In general, several works considered applying multiple algorithms to solve joint optimization problems as in^26,27^.

In the beginning, a modified K-means algorithm is used to provide suboptimal initial UAVs’ 3D location and GDs’ association. The modified K-means algorithm is used in the initialization phase due to its low complexity to reduce the number of framework iterations, hence, reducing the complexity of the proposed solution, which has been used similarly in^20^. The modification done on the K-means algorithm is fixing the centroids of the ground fixed base stations.

After that, the initial UAVs’ 3D locations and GDs’ associations are fed to a DRL algorithm, to optimize the UAVs’ 3D locations. Generally, different DRL algorithms are widely used in optimizing UAVs’ locations and trajectory problems, according to the environment and the application used^15–19,28–30^. Then the total GDs’ satisfaction index is checked, if there is an enhancement, the loop continues, and the new UAVs’ locations are fed to the regret learning algorithm. If there is no enhancement, the loop exits, the DRL UAVs’ locations output is neglected, and the last UAVs’ locations and GDs’ association are obtained to be the final optimized results.

After finding the optimum UAVs’ 3D locations, these locations are passed to a regret learning algorithm to optimize the GDs’ association. Due to its distributive nature, and since it is proven that it can reach a correlated equilibrium, the regret learning algorithm is widely used in solving GDs’ association problems^31–33^. After finding the optimum GDs’ association, the total GDs’ satisfaction is checked. If there is an enhancement, the loop continues, and the optimized GDs’ association solution is passed to the DRL algorithm. If there is no enhancement, the framework exits the loop, and the output from the regret learning algorithm is ignored while taking the last UAVs’ 3D location and GDs’ association as the optimized final result.

The proposed framework can operate in two modes, static UAVs mode, and mobile UAVs mode. In static UAVs mode, UAVs update their locations according to a predefined frequency of running the framework, which is more energy efficient^34–36^. In mobile UAVs mode, DRL algorithm run continuously when the framework is in the off periods, thus UAVs will update their locations continuously. Exploiting the full UAVs mobility will eventually results in better performance in terms of GDs satisfaction^37–39^. The frequency of running the framework is left as a parameter to the operator, since it depends on the mobility nature of the GDs in that specific hotspot area (i.e., frequency of changing their locations), and it depends on the minimum acceptable satisfaction index the operator would like to achieve.

The convergence of the proposed framework can be guaranteed by verifying its boundedness and monotonicity. Since the constraints (2) and (5) prevent the total sum of the satisfaction index *S* to grow unrestrictedly, the objective value denoted by $SER(A,\mathbf{X},\mathbf{Y},\mathbf{H})$ is bounded. For a fixed association $A^{(j)}$, the objective value $SER(A^{(j)},\mathbf {X^{(j+1)}},\mathbf {Y^{(j+1)}},\mathbf {H^{(j+1)}})$ is not less than $SER(A^{(j)},\mathbf {X^{(j)}},\mathbf {Y^{(j)}},\mathbf {H^{(j)}})$ in iteration $(j+1)$ by optimizing the UAVs locations. Similarly, by optimizing the GDs association considering the optimized UAVs locations, then $SER(A^{(j)},\mathbf {X^{(j+1)}},\mathbf {Y^{(j+1)}},\mathbf {H^{(j+1)}}) \le SER(A^{(j+1)},\mathbf {X^{(j+1)}},\mathbf {Y^{(j+1)}},\mathbf {H^{(j+1)}})$. Therefore, $SER(A^{(j)},\mathbf {X^{(j)}},\mathbf {Y^{(j)}},\mathbf {H^{(j)}}) \le SER(A^{(j+1)},\mathbf {X^{(j+1)}},\mathbf {Y^{(j+1)}},\mathbf {H^{(j+1)}})$, and the framework is convergent^40–43^.

### Deep reinforcement learning

In this section, we exploit the DRL algorithm, to solve the optimization problem of finding the UAVs’ optimum locations to maximize the SER. In the beginning, each UAV is considered to be an agent. Generally, an agent in reinforcement learning can learn its optimal policy by interacting with the environment, to maximize the expected cumulative reward over time (i.e., to learn the optimal action sequence that leads to the defined goal)^44^. The agent observes its current state, then it takes an action, and an immediate reward is received along with the next state. This process is repeated, and it is used by a reinforcement learning algorithm to adjust the agent’s policy until it approaches the optimal policy^45^.

A reinforcement learning agent is typically modeled as a finite Markov Decision Process (MDP) if the state and action spaces are finite. For multiple agents with full observability (i.e., an agent can observe its state and other agents’ states), MDP can be extended to stochastic games^46^. For multiple agents with partial observability, a Decentralized Partially Observable Markov Decision Process (Dec-POMDP) can be considered^46^. A Dec-POMDP is characterized by the tuple $(\mathcalligra{\scriptstyle U} \hspace{000.1cm},\mathcalligra{\scriptstyle S} \hspace{000.1cm},\mathbb {A},p_{s\left( t\right) ,s(t+1)},r(t),\mathbb {O},\mathcalligra{\scriptstyle O} \hspace{000.1cm})$, where $\mathcalligra{\scriptstyle U} \hspace{000.1cm}$ is the set of agents (i.e., UAVs); $\mathcalligra{\scriptstyle S} \hspace{000.1cm}$ denotes the finite set of the state space, $\mathbb {A}$ is the joint action space of the agents concatenated by$\mathbb {A}\triangleq \mathcalligra{\scriptstyle A} \hspace{000.1cm}_1\times \mathcalligra{\scriptstyle A} \hspace{000.1cm}_2\times \ldots \times \mathcalligra{\scriptstyle A} \hspace{000.1cm}_U$, $p_{s_ts_{t+1}}$ is the transition probability from state $s(t)\in \mathcalligra{\scriptstyle S} \hspace{000.2cm}$ at time step *t* to state $s(t)\in \mathcalligra{\scriptstyle S} \hspace{000.2cm}$ after taking action $a(t)\in \mathcalligra{\scriptstyle A} \hspace{000.2cm}$, and *r*(*t*) denotes the agent’s immediate reward at time step *t* after performing action $a(t)\in \mathcalligra{\scriptstyle A} \hspace{000.1cm}$, $\mathbb {O}$ denotes the joint observation space concatenated by the observation of the *k*th agent as $\mathbb {O}\triangleq \mathbb {O}_1\times \mathbb {O}_2\times \ldots \times \mathbb {O}_U$;$\mathcalligra{\scriptstyle O} \hspace{000.1cm}:\mathcalligra{\scriptstyle S} \hspace{000.1cm}\times \mathbb {A}\times \mathbb {O}\rightarrow \left[ 0,1\right]$ is the observation function The details of the components of the Dec-POMDP are described as follows:

- State: the state of the environment at time *t* is the 3D locations of the UAVs, and it can be expressed as:
  16 $$\begin{aligned} s(t)=\{x_1(t),x_2(t),\ldots ,x_U(t),\ y_1(t),y_2(t),\ldots ,y_U(t),h_1(t),h_2(t),\ldots ,h_U(t)\} \end{aligned}$$
- Action: the agents’ actions are the movement of the UAVs in the possible directions. The actions of the *u*th UAV can be expressed as
  17 $$\begin{aligned} a_u(t)=\{Up,\ down,\ right,\ left,\ forward,\ backward,\ stay\} \end{aligned}$$
- Observation: Since we consider agents partial observability, each UAV can only observe its state (i.e., 3D location). The UAV’s u observation can be denoted and expressed by $o_u\left( t\right) =\{x_u\left( t\right) ,y_u\left( t\right) ,h_u(t)\}$. For scenarios where full observability is considered, each UAV can observe its own location, and other UAVs’ locations as well.
- Reward: Since the objective is to maximize the total GDs’ satisfaction while minimizing the UAVs’ energy consumption, the reward function for UAV u is calculated by $r_u\left( t\right) =\frac{\sum _{i,k}{A_{i,k}S_i}}{\sum _{k\in \mathcalligra{\scriptstyle U} \hspace{000.1cm}} E_k}$

The DQN algorithm utilizes a target network alongside an online network to stabilize the overall network performance, which iteratively can find the optimal state action value function $\mathcalligra{\scriptstyle Q} \hspace{000.1cm}^*(s,a)$ for all state-action pairs. The Q-network updates its weights to minimize the loss function defined as^47^:

18 $$\begin{aligned} L_t(\theta )=E_{s(t),a(t),r(t)}\left[ {(y_t^{DQN}-\mathcalligra{\scriptstyle Q} \hspace{000.1cm}_t^\pi \left( s(t),a(t);\theta \right) )}^2\right] \end{aligned}$$

Where $y_t^{DQN}=r(t)+\gamma \max _{a}{\mathcalligra{\scriptstyle Q} \hspace{000.1cm}_t^\pi \left( s(t+1),a;\theta ^-\right) }$, such that $\theta ^-$ represents the weights of the target network, and it is updated by the online network weights $\theta$ every fixed number of steps. Since the same samples are used in selecting and evaluating the actions, this more likely leads to selecting over-estimated values. Thus, DDQN was introduced to solve this problem by replacing $y_t^{DQN}$ by $y_t^{DDQN}=r(t)+\gamma \mathcalligra{\scriptstyle Q} \hspace{000.1cm}_t^\pi (s(t+1),\max _{a}{\mathcalligra{\scriptstyle Q} \hspace{000.1cm}_t^\pi \left( s(t+1),a;\theta \right) }\theta ^-)$. This means that $\theta$ is used in the online network and value estimation in the target network as well, however, $\theta ^-$ is used to evaluate fairly the value of this policy.

**Algorithm 1:**
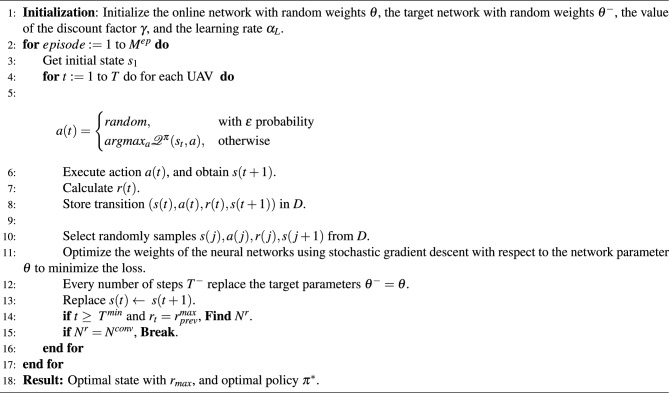
DDQN algorithm with experience replay for UAV 3D deployment

Algorithm 1 illustrates the DDQN algorithm for UAVs’ 3D deployment. After initialization, for every episode until the maximum number of episodes $M^{ep}$, and in every step *t*, action *a*(*t*)is selected according to the $\epsilon$-greedy policy, which represents the trade-off between exploration and exploitation (lines 1-5). After action execution and moving to the new state, the reward is calculated, and the whole experience is stored in the replay memory (lines 6-8). Then, random samples are selected from the replay memory to optimize the weights $\theta$. After a specified number of steps $T^-$, target network parameters $\theta ^-$ are updated with the online network parameters $\theta$ (lines 9-12). In the learning phase, the optimal policy is obtained by executing steps until *T*, however, in the testing phase, the optimal state is obtained by executing steps until $r(t)=r_{prev}^{max}$ is repeated $N^{conv}$ times, and the number of steps is more than $T^{min}$ (lines 13-17).

To express the computation complexity of the DDQN algorithm, the training phase and the testing phase should be differentiated. In the training phase, in each time step for each agent, the computation complexity can be expressed by $O(N_0^{neu} N_1^{neu}+\sum _{(\iota =1)}^{I}N_\iota ^{neu} N_{(\iota +1)}^{neu})$^48^. Where, *I*, $N_0^{neu}$, and $N_\iota ^{neu}$ denote the number of layers, the size of the input layer, and the size of layer $\iota$. Considering $M^e_p$ number of episodes, *T* number of iterations for each trained model under different number of GDs until convergence, the total computation complexity is $O\left( UM^e_p T\left( N_0^{neu} N_1^{neu}+\sum _{(\iota =1)}^{I}N_\iota ^{neu} N_{(\iota +1)}^{neu}\right) \right)$^48–50^. It should be noted that the training phase is executed offline, due to the high training computation complexity. The computation complexity is relaxed in the testing phase so that it can be calculated by $O(|\mathcalligra{\scriptstyle S} \hspace{000.1cm}| \times |\mathbb {A}|)$^48^.

### Correlated equilibrium and regret-based learning

To solve the optimization problem of finding the optimal GDs’ association with the available base stations, to maximize the satisfaction index, we adopt the widely known regret learning algorithm. This learning algorithm is based on the notion of regret matching^51^. It has been proved that using the procedures in this algorithm will result in a popular notion of rationality called the Correlated Equilibrium (CE)^51^. The notion of CE is a generalization of the Nash equilibrium, where it is an optimality concept that has been proven to exist for every finite game with its payoffs bounded^52^. It was introduced by the Nobel prize winner Robert J. Aumann^52^, in 1977. The idea of CE is that the GD’s strategy profile is chosen randomly according to the probability distribution, where each GD has no benefit of choosing any other probability distribution, and it’s in his best interest to conform with this strategy^31^.

In this context, the proposed finite game is denoted by $\mathcalligra{\scriptstyle J} \hspace{000.2cm}=(\mathcalligra{\scriptstyle G} \hspace{000.1cm},\ \Sigma ,\ \Phi )$, such that $\mathcalligra{\scriptstyle G} \hspace{000.1cm}$ is the set of GDs, $\Sigma =\mathrm {\Sigma }_1\times ,,,\times \mathrm {\Sigma }_N$ is set of joint strategies for all GDs, while $\mathrm {\Sigma }_i\subset \mathcalligra{\scriptstyle K} \hspace{000.1cm}$ is the set of finite strategies for GD *i*, and $\Phi$ is the set of utility functions for all GDs, which is represented by the satisfaction index each GD, such that $\mathrm {\Phi }_i=\ S_i$ is the utility function of GD *i*. A probability distribution $\psi$ is a correlated equilibrium of $\mathcalligra{\scriptstyle J} \hspace{000.2cm}$, if for every player $i\in \mathcalligra{\scriptstyle G} \hspace{000.1cm}$, and a pair of actions $m_i,m_i^\prime \in \mathrm {\Sigma }_i$, it holds that:

19 $$\begin{aligned} \sum _{m_{-i}\in \mathrm {\Sigma }_{-i}}{\psi (m_i,m_{-i})\left( \mathrm {\Phi }_i\left( m_i^\prime ,m_{-i}\right) -\mathrm {\Phi }_i\left( m_i,m_{-i}\right) \right) }\le 0 \end{aligned}$$

This implies that for GD *i*, choosing action $m_i^\prime$ will not produce a better expected payoff compared to action $m_i$. Thus, CE models the correlation between GDs’ actions, while in Nash equilibrium, GDs would choose their actions independently.

The regret matching algorithm exploits the notion of CE. The main idea of the algorithm is that the probability of choosing a strategy should be proportional to the “regret” for not having chosen other strategies. To define the probability distribution that yields this probability, we first define the regret of player *i* for not playing strategy $m_i$ instead of $m_i^\prime$ at time *t* is^31^:

20 $$\begin{aligned} \rho _i^t\left( m_i,m_i^\prime \right) \triangleq \max (D_i^t\left( m_i,m_i^\prime \right) ,0) \end{aligned}$$

Where $D_i^t\left( m_i,m_i^\prime \right)$ represents the payoff for player *i* if he had played action $m_i^\prime$ instead of $m_i$ every time in the past, and it can be calculated as follows^31^:

21 $$\begin{aligned} D_i^t\left( m_i,m_i^\prime \right) \triangleq \frac{1}{t}\sum _{T\le t}\left( \mathrm {\Phi }_i^T\left( m_i^\prime ,m_{-i}\right) -\mathrm {\Phi }_i^T\left( m_i,m_{-i}\right) \right) \end{aligned}$$

Thus, the probability distribution of player *i* chooses an action at time *t* is^31^:

22 $$\begin{aligned} \psi _i^{t+1}\left( m_i\right) = {\left\{ \begin{array}{ll} \frac{1}{\mu }\rho _i^t(m_i,m_i),& m_i\ne m_i\\ 1-\sum \limits _{m_i\in \Sigma _i,m_i\ne m_i}\psi _i^{t+1}(m_i),& m_i=m_i \end{array}\right. } \end{aligned}$$

Where $\mu >2MG$ is a constant that guarantees $\psi _i^{t+1}\left( m_i\right) >0$ at $m_i=m_i^\prime$, and *G* is the upper bound of $\left| \phi (m_i)\right|$ for all $m_i\in \mathrm {\Sigma }_i$^31^. At $t=1$ the initial probability is distributed uniformly over the set of possible actions.

It can be noted that the player’s *i* probability of choosing actions $m_i$ is a linear function of the regrets. It is also proven in^31^ that the empirical distribution ${\bar{z}}_t$ of joint actions $\mathbf{m}$ of all players until *t*:

23 $$\begin{aligned} {\bar{z}}_t\left( \mathbf{m}\right) =\frac{1}{t}N(t,\mathbf{m}) \end{aligned}$$

Where $N(t,\mathbf{m})$ denotes the number of periods before *t* that action $\mathbf{m}$ has been chosen, converges almost surely (with probability 1) to the set of CE in the regret matching algorithm.

**Algorithm 2:**
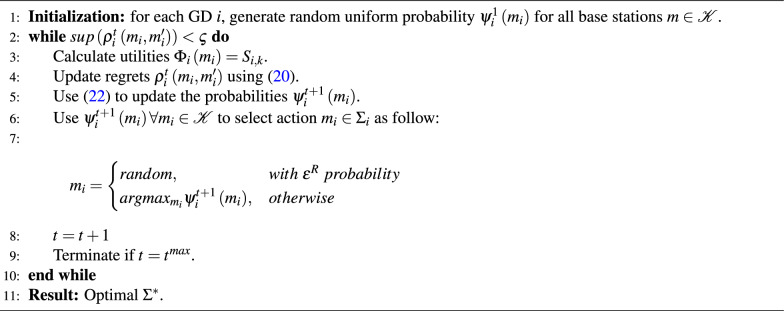
Regret-based learning algorithm for GDs to base stations association

Algorithm 2 shows the regret-based learning algorithm used to find the optimal $\Sigma ^*$ that can be mapped to $A^*$. In the beginning, the utilities $\mathrm {\Phi }_i\left( m_i\right) =S_{i,k}$ is calculated, then the regrets $\rho _i^t\left( m_i,m_i^\prime \right)$ are updated (lines 1 to 4). After that, for each GD *i*, the probability distribution $\psi _i^{t+1}\left( m_i\right)$ is calculated, such that strategy $m_i$ is selected based on $\varepsilon ^R$-greedy policy (lines 5,6)^51^. The previous lines are repeated until $sup\left( \rho _i^t\left( m_i,m_i^\prime \right) \right) <\varsigma$, where $\varsigma$ should be properly selected as in^51^.

The computation complexity of the regret-based learning algorithm can be expressed by $\mathcalligra{\scriptstyle O} \hspace{000.1cm}(\frac{1}{\sqrt{t}})$ for regret expectations $E\left[ \rho _i^t\left( m_i,m_i^\prime \right) \right]$^51^. In addition, the convergence time is asymptotically bounded by $\frac{1}{\varsigma ^2}$, such that $t_{conv}=\Omega (\frac{1}{\varsigma ^2})$, which means that to speed up the convergence, $\varsigma$ should be selected sufficiently small^33^.

## Performance evaluation and discussion

In this section, the performance of the proposed work is evaluated in terms of the proposed framework, system architecture, and different system parameters.

### Simulation setup

In general, a Multi-RAT HetNet ground environment is considered, where it comprises an MBS with 1000*m* coverage, and two small base stations (i.e., one FBS, and one WAP) deployed to cover a hot spot area of $150m\times 275m$. Multiple aerial base stations are considered to serve the hot spot area as well, such that two UAVs are considered, each carrying an FBS and a WAP. Except for hovering, and to minimize the UAVs’ power consumption, each UAV can move 18*m* in any direction according to $\mathcalligra{\scriptstyle A} \hspace{000.1cm}$ at each time step, where the time step is considered to be 1 second, considering $V_{mr}=18m/s$. For hovering, UAV’s velocity is $0\ m/s$, and it stays at its location for the 1 second time step. Moreover, for path loss, $\eta _{LoS}= 3dB$ and $\eta _{NLoS}=23dB$ for LoS and NLoS path loss coefficients are considered, respectively, along with a path loss exponent $\alpha \ = 2$. While $a = 11.95$ and $b =0.14$ are considered for urban environments^20^. An additive noise power of $-174dBm/Hz$ is considered for GDs^22^. The rest of the simulation parameters are summarized in Table 3.

**Table 3:** Simulation parameters.

WLAN parameters	Values	LTE parameters	Values
Bandwidth	20 MHz	LTE bandwidth	20 MHz
$f^{WLAN}$	2.4 GHz	Channel bandwidth	180 kHz
WLAN technology	802.11n	Transmit power of MBS	46 dBm
Transmit power of WAP	200 mW	Transmit power of FBS	20 dBm
Uplink packets generation rate	0.0004 Packets/slot	Target uplink SNR for GDs	10 dB
$\textrm{SINR}_k^{thr}$ for WUBS	1 dB	$\textrm{SINR}_k^{thr}$ for FUBS	$-4.46$ dB
Minimum contention window(*W*)	16	DDQN parameters	Values
Maximum number of re-transmissions ($\mu$)	6	$M^{ep}$	5000
Slot time	$9 \mu s$	*T*	200
DIFS	$50 \mu s$	$\varepsilon$	$5\times {10}^{-5}$
SIFS	$10 \mu s$	$\gamma$	0.9
*D*	1500 bytes	$\alpha _L$	$2\times {10}^{-8}$
ACK	160 bits	Regret learning parameters	Values
RTS	208 bits	$t^{max}$	50
CTS	160 bits	$\varepsilon ^R$	0.1

### Performance evaluation criteria

In the beginning, the performance of the proposed multi-agent DRL algorithm for maximizing the SER by optimizing the UAVs’ 3D locations is compared to other benchmark algorithms, considering different training and observation techniques. In particular, since the DDQN algorithm performs better than other discrete value-based algorithms^53^, a comparison between the DDQN discrete algorithm and the Deep deterministic policy gradient (DDPG) algorithm has been conducted. DDPG algorithm is an off-policy that uses an actor-critic approach considering a continuous action space to solve continuous problems, and it is widely used in optimizing UAVs’ trajectory and location in different applications^54–57^. In this regard, the action space of the DDPG algorithm for each UAV $u\in \mathfrak {U}$ is denoted by $\mathcalligra{\scriptstyle A} \hspace{000.1cm}^{DDPG}=\left\{ \chi ^{DDPG},\vartheta ^{DDPG},\phi ^{DDPG}\right\}$, where $\chi ^{DDPG}\in [0,18], \vartheta ^{DDPG}\in [0,2\pi ]$, and $\phi ^{DDPG}\in [0,2\pi ]$ are the flight distance in meters, the flight angle in the X-plane in radians, and the flight angle in the Z-plane in radians, respectively. In every time step, each UBS moves with $V_{mr}$ to perform its action, then it holds its location until the end of the time step.

Moreover, the DDQN and DDPG algorithms are compared considering centralized control training, decentralized coordination training, the agents’ complete information, and incomplete information about the actual model. Specifically, for complete information, each agent chooses its action with the knowledge of other agents’ actions, locations, and rewards. However, for incomplete information, each agent chooses its action without perfect knowledge of other agents’ actions, locations, and rewards. It should be noted that in the testing phase, agents collect their experiences individually, i.e., they work with decentralized coordination.

It is worth noting that, DDQN has a critic network composed of an observation path, an action path, and a common path. The observation path has an input layer of size 3 for incomplete information and of size 6 for complete information to catch the other UAV 3D location, followed by 4 fully connected layers of size 32, a scaling layer, and a ReLU layer. The action path has an input layer of size 1, 4 fully connected layers of size 32, and a ReLU layer. The common path has an addition layer to add the observation and action paths, followed by 4 fully connected layers of size 32, each of which is followed by a ReLU layer. For DDPG, the critic network has the same observation path. The action path has an input layer of size 3, 4 fully connected layers of size 32, and a Tanh layer. The common path is the same as in DDQN, but with replacing the ReLU with the Tanh layer. Since DDPG has an actor-network as well, this network has the same input layer as the observation layer of the critic network, followed by 5 fully connected layers of size 32, each of which is followed by a Tanh layer.

After that, the performance of the proposed work is evaluated in terms of system architecture with previous works, where the performance of deploying both a WAP, and an FBS on each UAV is compared by both scenarios of deploying only an FBS on each UAV, as in^58–60^, and deploying only a WAP on each UAV, as in^8–10^, such that they are abbreviated by “LTE-Wi-Fi UAVs”, “LTE UAVs”, and “Wi-Fi UAVs”, respectively, for the rest of the discussion. For comparison fairness, in LTE UAVs and Wi-Fi UAVs, a number of WAPs and FBSs equivalent to the disseminated WAPs and FBSs from the LTE-Wi-Fi UAVs, respectively, are added to the Multi-RAT HetNet ground environment to compensate the deployment of two base stations on each UAV in our proposed deployment scenario.

The locations of the added base stations are optimized as in^61^ to compensate for the degree of freedom offered in our proposed scenario. In this comparison, multiple various network performance metrics are considered, starting with the GD’s average satisfaction ratio, GD’s average downlink achieved data rate, GD’s average uplink power consumption, GD’s Jain’s fairness index, GD’s average outage probability, which is defined as the GD’s probability of not achieving the reference downlink rate, and ending with the measuring the number of framework iterations.

Eventually, the performance of the proposed work is evaluated at different average GD’s requirement settings, where the Cumulative Distribution Function (CDF) of the GD’s average satisfaction index, GD’s uplink power consumption, and average GDs’ downlink sum rate.

### Results and discussion

**Fig. 4:**
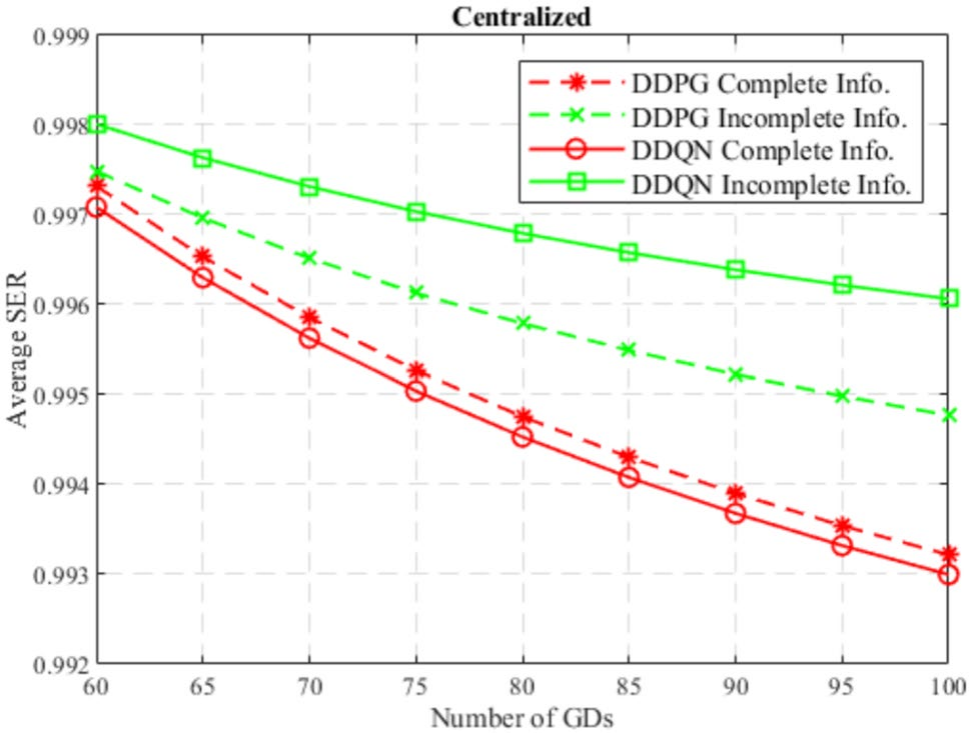
SER for centralized DDQN and DDPG.

**Fig. 5:**
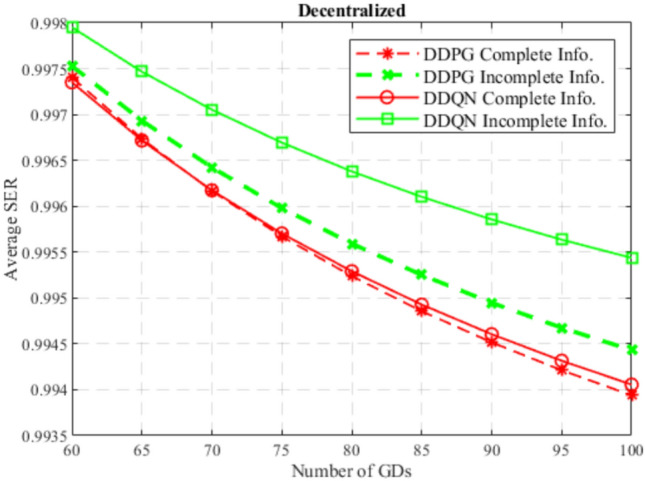
SER for decentralized DDQN and DDPG.

**Fig. 6:**
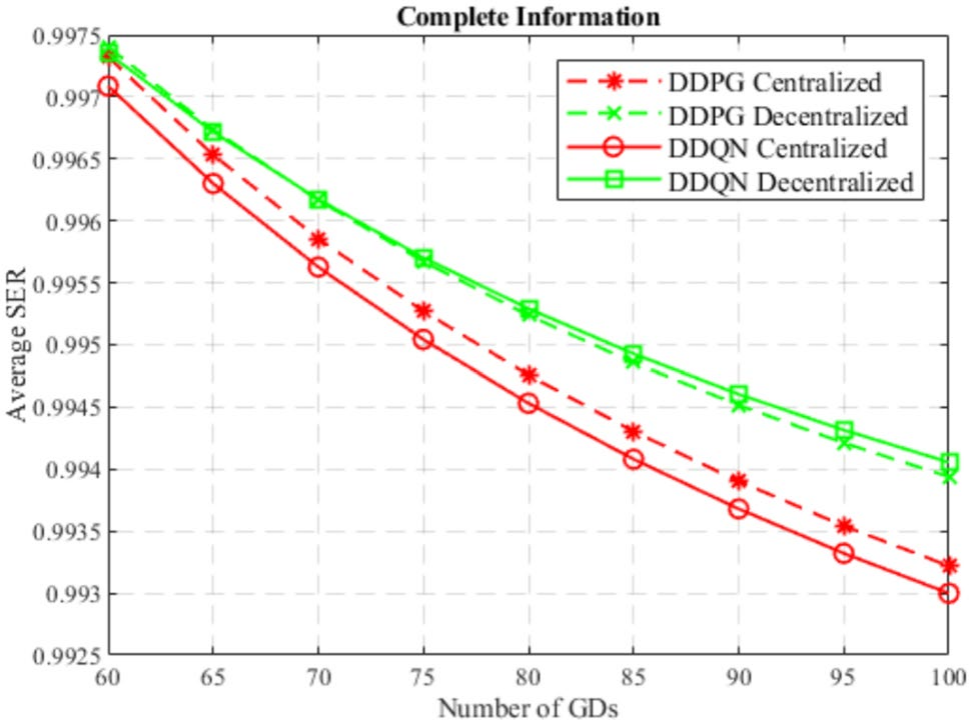
SER for complete information on DDQN and DDPG.

**Fig. 7:**
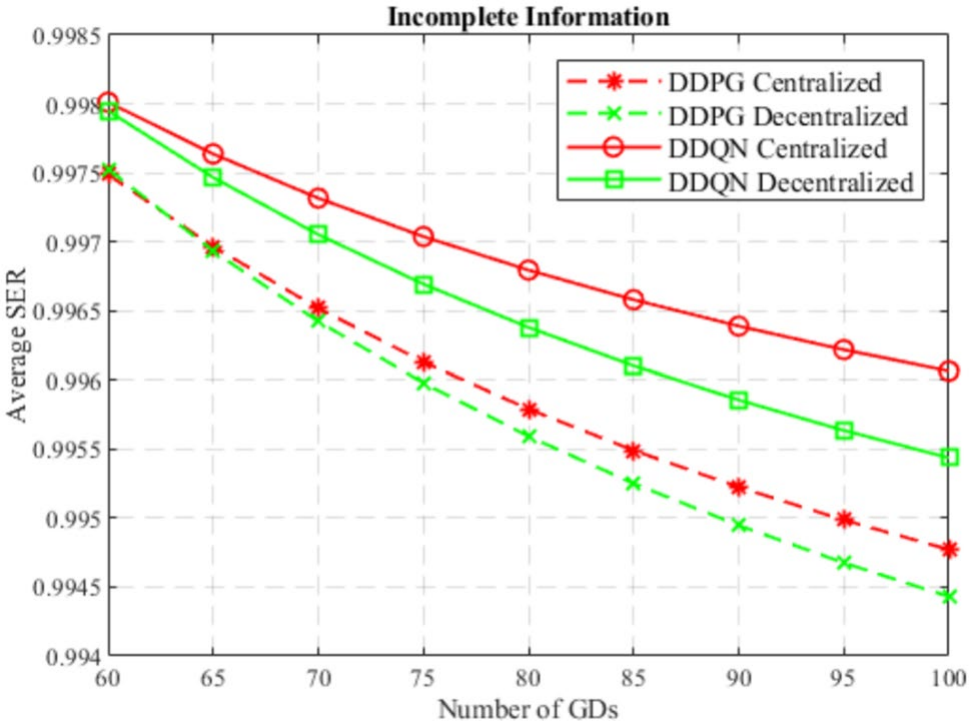
SER for incomplete information on DDQN and DDPG.

At the beginning, the performance of the DDQN algorithm is compared to the DDPG algorithm considering centralized and decentralized training approaches, and considering complete and incomplete model information as well. In Figs. 4 and 5, DDQN and DDPG algorithms are compared in terms of average SER with complete and incomplete information considering centralized training, and decentralized training, respectively. While Figs. 6 and 7 compare DDQN and DDPG algorithms with centralized and decentralized training considering complete information, and incomplete information, respectively.

In general, from these figures, it can be observed that the average SER decreases with the increase in the number of GDs. This is due to the decrease in the achieved downlink data rate per GD and the increase in per-GD uplink power consumption, since the GD’s share of bandwidth decreases in LTE, and contention increases in Wi-Fi, leading to a decrease in the satisfaction ratio, decreasing the SER accordingly.

Moreover, it can also be noticed that in a centralized training approach, DDPG outperforms DDQN while using complete information, while DDQN surpasses DDPG using incomplete information. This is because the DDPG algorithm works with continuous action space, which offers precise control, and when combined with the full observability of other agents’ actions, this enables optimal positioning relatively. However, in an incomplete information approach, where agents rely on their partial observation of the environment and other agents, the DDQN algorithm with its discrete nature, facilitates the exploration encouraged by incomplete information and offers simplicity in decision making, which provides robust and adaptable strategies to cope with the uncertainty introduced by incomplete information.

In general, the DDQN algorithm shows better results than the DDPG algorithm due to its simplicity in decision making, robustness to uncertainty, and better generalization. Besides, the downlink GD data rate and the uplink GD power consumption are discretized since MCS schemes are adopted in LTE and Wi-Fi networks, which makes the environment tend to fit into discrete action and state space models. Also, the DDPG algorithm suffers from less robustness to uncertainty, complex decision making, and difficult exploration in uncertain conditions, due to the continuity nature of its action space. However, for complete information approaches and a centralized training approach, where the DDPG algorithm excels due to the precise control, leveraged by the full observability, and exploiting the high coordination offered by the centralized training, where agents share the same critic and policy.

It can also be observed that the DDPG algorithm outperformed the DDQN algorithm considering complete information, only in the centralized training approach, while in decentralized training DDQN algorithm performed better. This is because centralized training develops highly coordinated strategies by leveraging the shared experiences of all agents, which meets the needs of the continuous nature of the DDPG algorithm when a complete information approach is considered. In decentralized training, this coordination is not available, which encourages individual learning and adaptability, which is fulfilled by the simplicity of the DDQN algorithm.

Furthermore, it can be noticed that in general, incomplete information approach outperform complete information approach, regardless of the algorithm used or the training. This is because incomplete information encourages broader exploration discovering more effective actions, and prevents falling into suboptimal strategies, leading to more robust and generalized strategies. On the other hand, complete information may lead to overfitting, where agents learn strategies that rely on perfect knowledge and full observability of the environment, which is less robust to unexpected variations or untrained situations during testing. Not to mention the fact that complete information is hard to achieve in real-world.

In the end, the DDQN algorithm with incomplete information using a centralized training approach achieves the best performance of all others. This is because centralized training allows sharing experiences between agents, which excels the exploration in a simpler and discrete environment, to obtain optimal and generalized strategies, that are influenced by the uncertainty offered by the incomplete information.

To summarize, these comparisons highlight the importance of selecting the best algorithm, training approach, and information availability. Also, they illustrate the complex interplay between the DRL algorithm, its training approach, and the information availability, that can be efficiently fit in the proposed environment. The optimal combination is the DDQN algorithm combined with an incomplete information that excels in centralized training, which provides robust and generalized policies that can cope efficiently with the dynamic nature of the proposed environment.

**Fig. 8:**
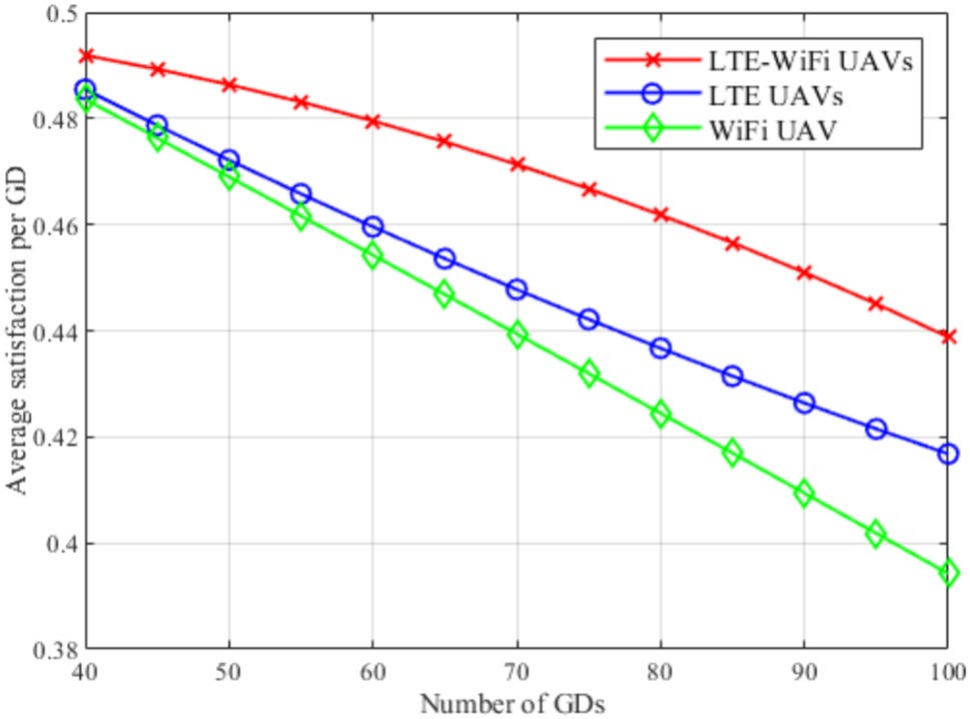
Average satisfaction between different deployment scenarios.

**Fig. 9:**
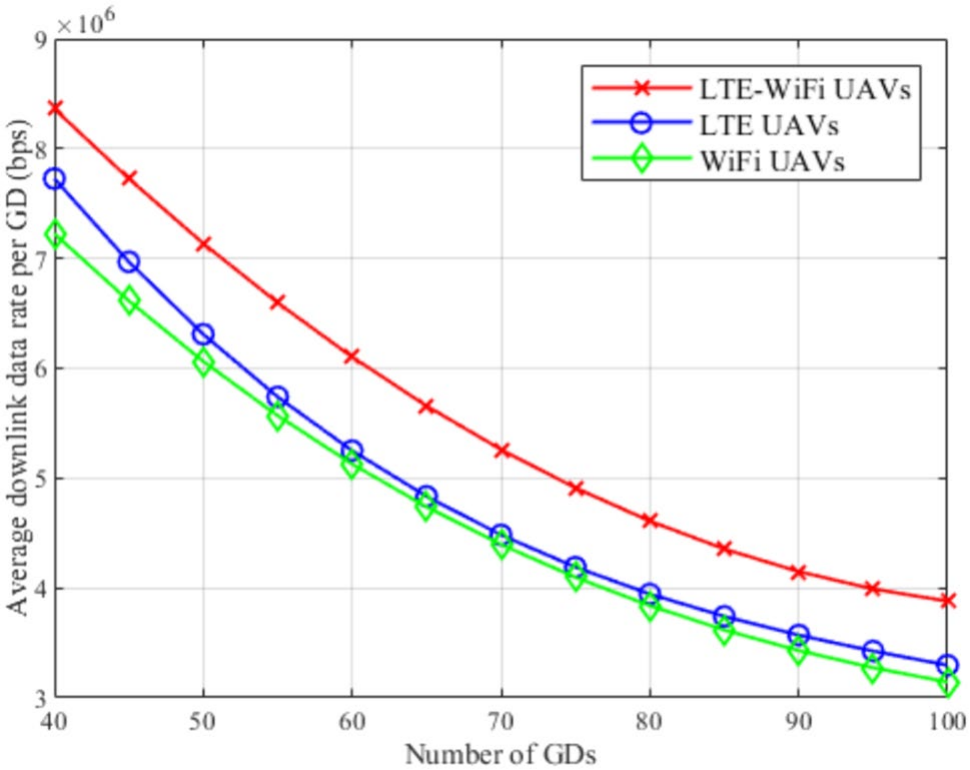
Average downlink data rate between different deployment scenarios.

**Fig. 10:**
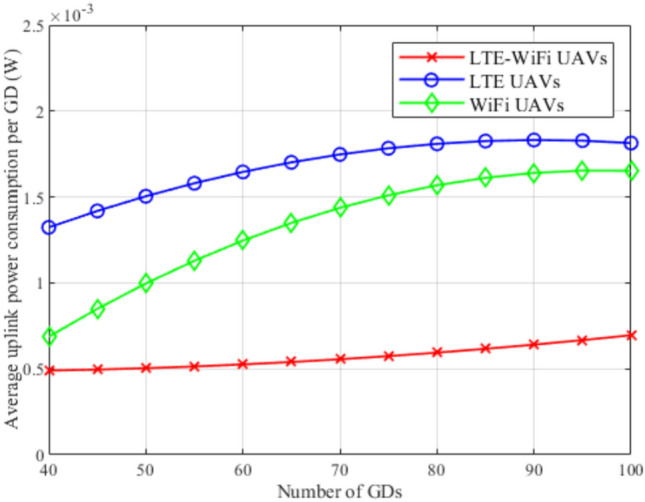
Average uplink power consumption between different deployment scenarios.

**Fig. 11:**
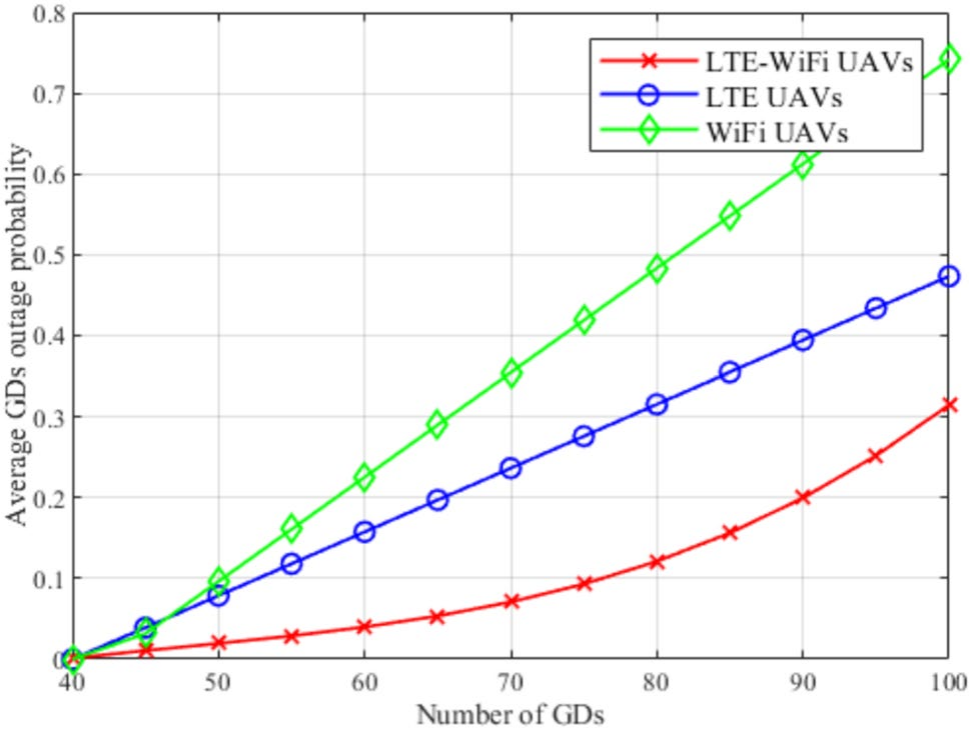
Average outage probability between different deployment scenarios.

**Fig. 12:**
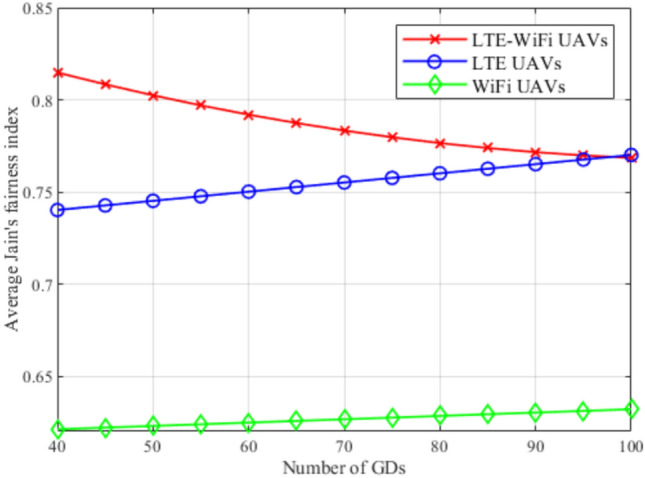
Jain’s fairness index between different deployment scenarios.

**Fig. 13:**
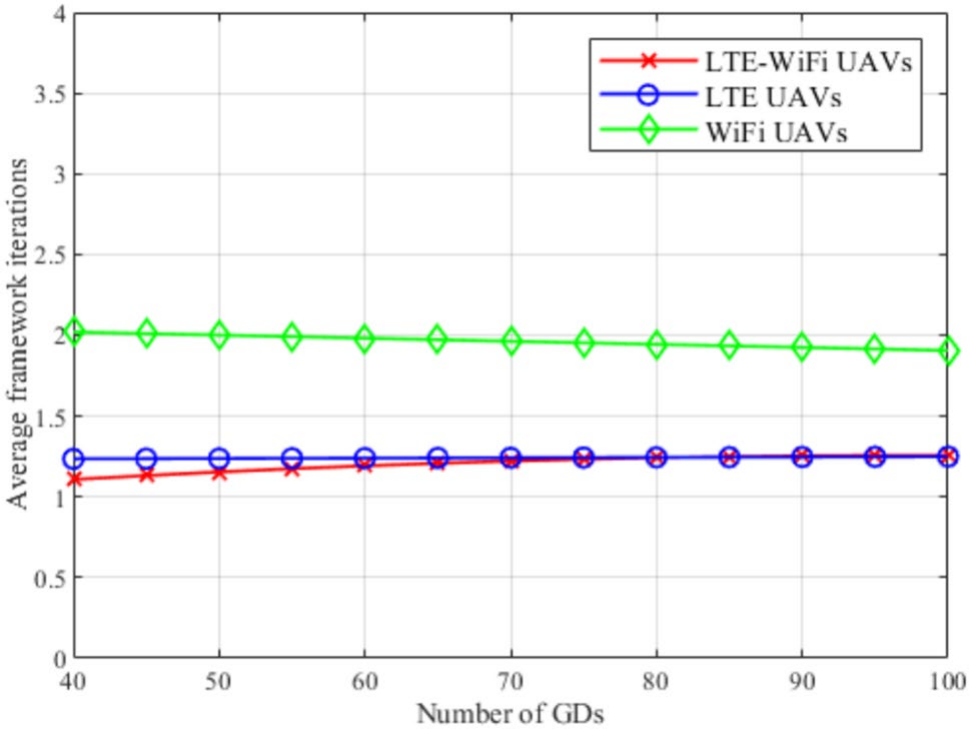
Framework iterations between different deployment scenarios.

Figures 8, 9, 10, 11, 12, and 13, evaluate and compare the proposed coupled LTE-Wi-Fi UAVs, where the framework optimizes the location of coupled LTE and Wi-Fi base stations for each UAV, and the decoupled LTE UAVs and Wi-Fi UAVs deployment scenarios, where Wi-Fi base stations and LTE base stations are decoupled from UAVs, respectively, and their locations are optimized and fixed on the ground before running the framework. Therefore, it is a tradeoff between increasing the degree of freedom at the expense of coupling LTE to Wi-Fi base stations on UAVs, and between decoupling LTE and Wi-Fi base stations deployed on UAVs, at the expense of optimizing and fixing the location of ground base stations before running the framework. Taking into consideration that, LTE-Wi-Fi UAVs are a cost-effective solution compared to other scenarios, where two base stations can be deployed on each UAV, thus a smaller number of UAVs can be evoked upon request due to sudden traffic congestion, or disaster. It is worth noting that these results were obtained at $R^{ref}=3$ Mbps.

In Fig. 8, the average satisfaction index per GD is evaluated between LTE-Wi-Fi UAVs, LTE UAVs, and Wi-Fi UAVs, while increasing the total number of GDs in the system. It can be observed that, in general, the average satisfaction index per GD decreases by increasing the number of GDs, which was justified in the analysis of Fig. 4. Also, it can be noticed that LTE-Wi-Fi UAVs deployment exceeds other deployments. This illustrates the effectiveness of increasing the degree of freedom over decoupling the Multi-RAT UAVs in terms of satisfaction index, and converting them into single RAT UAVs, while optimally deploying other decoupled RAT base stations on the ground. Moreover, LTE UAVs deployment surpasses Wi-Fi UAVs deployment because LTE base stations suffer from higher interference, especially from the MBS, so deploying them on UAVs, and optimizing their locations according to the changes in the environment will be more effective, compared to deploying Wi-Fi base stations on UAVs.

Figure 9 compares LTE-Wi-Fi UAVs, LTE UAVs, and Wi-Fi UAVs deployments in terms of the average downlink data rate per GD while increasing the number of GDs. It can be noticed that LTE-Wi-Fi UAVs achieves the best performance, which is a normal reflection of the LTE-Wi-Fi UAVs outperformance in terms of satisfaction index. It can be shown that increasing the number of GDs decreases the per-GD downlink data rate, despite the used deployment, which is justified by the increase in the network load.

In Fig. 10, LTE-Wi-Fi UAVs, LTE UAVs, and Wi-Fi UAVs deployments are compared in terms of the average uplink power consumption per GD while increasing the number of GDs. In this figure, the LTE-Wi-Fi UAVs has better performance compared to other deployments. Along with Fig. 10 observations, these observations reflect the effectiveness of optimizing the location of coupled LTE and Wi-Fi base stations on each UAV. It can also be noticed that Wi-Fi UAVs achieves superior performance compared to LTE UAVs, despite achieving worse performance in terms of satisfaction index. This is because the objective function is to maximize GDs’ satisfaction in general. In LTE UAVs, location is optimized to exploit reducing the interference, hence increasing the downlink data rate, which has higher effects on the satisfaction index, since GDs suffers from higher uplink power consumption when connecting to LTE base stations compared to connecting to Wi-Fi base stations in general. On the contrary, in Wi-Fi UAVs, the interference in from LTE base stations remains the same, hence, Wi-Fi UAVs favors optimizing their locations to achieve better uplink power consumption, where they can increase their performance in terms of satisfaction index. The increase in average per-GD power consumption with the increase in the number of GDs also can be observed, which is justified by the increase in LTE network congestion, and the increase in contention for the Wi-Fi base stations.

Although outage probability is not considered in the proposed objective function, it is used as a performance metric to determine which deployment scenario will provide the greater number of GDs with the required downlink data rate $R^{ref}$. It can be observed in Fig. 11 that LTE-Wi-Fi UAVs achieve the lowest outage probability compared to other deployments. When these observations are combined with the observations of Fig. 9, where LTE UAVs and Wi-Fi UAVs achieve almost the same average downlink data rate per GD, this implies that in Wi-Fi UAVs deployment, more GDs achieve data rates less than $R^{ref}$, while other GDs achieve relatively high data rates, so that Wi-Fi UAVs deployment can achieve the same average per-GD data rate compared with LTE UAVs deployment.

In Fig. 12, Jain’s fairness index performance metric is used to compare the fairness of the different deployments. It can be observed that LTE-Wi-Fi UAVs deployment also performs better than other deployments, despite that fairness is not considered in the proposed objective function. This implies that the GDs’ downlink data rates are relatively close to each other. This comes from the greedy distributed nature of the regret learning algorithm, where each GD tries to associate with the best base station that can serve it. Which means that optimizing the LTE-Wi-Fi UAVs locations offers better associations compared with optimizing decoupled LTE and Wi-Fi UAVs.

Figure 13 shows the effect of using the different deployments on the average number of framework iterations, by increasing the number of GDs. It can be shown that the LTE-Wi-Fi, and LTE UAVs deployments almost achieve the same average number of framework iterations, with slightly better performance for the LTE-Wi-Fi UAVs. Both scenarios achieve better performances compared with Wi-Fi UAVs. This implies that the LTE-Wi-Fi, and LTE UAVs deployments almost achieve the same performance in terms of complexity and computational cost, and better performance compared to Wi-Fi UAVs.

**Fig. 14:**
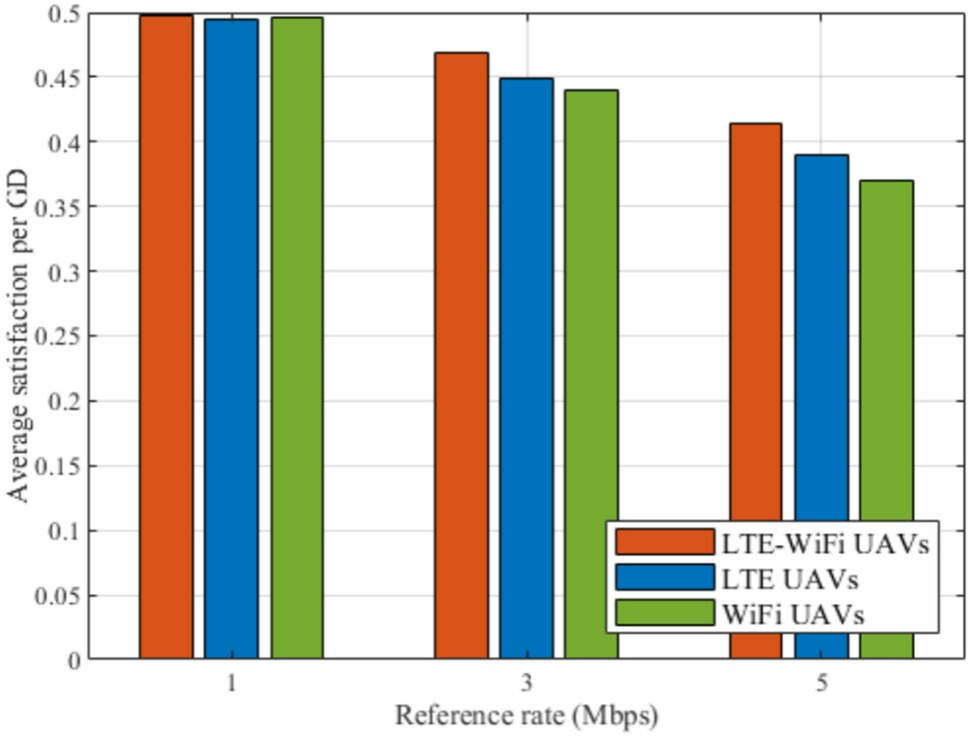
Average satisfaction per GD at different reference rates.

**Fig. 15:**
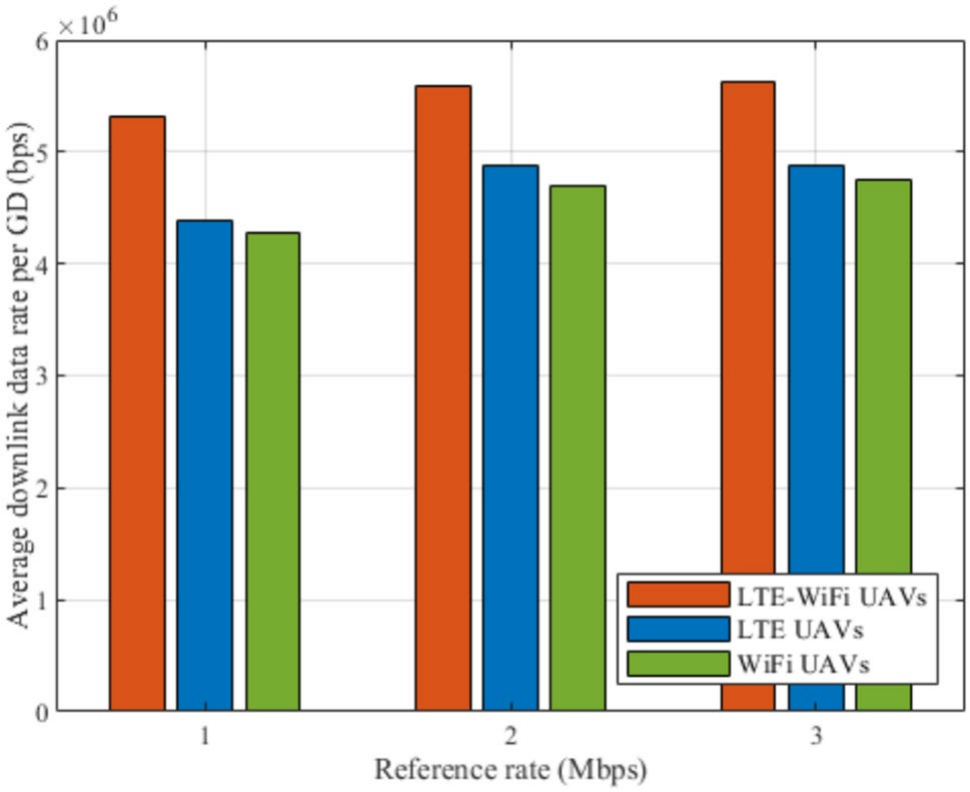
Average downlink data rates per GD at different reference rates.

**Fig. 16:**
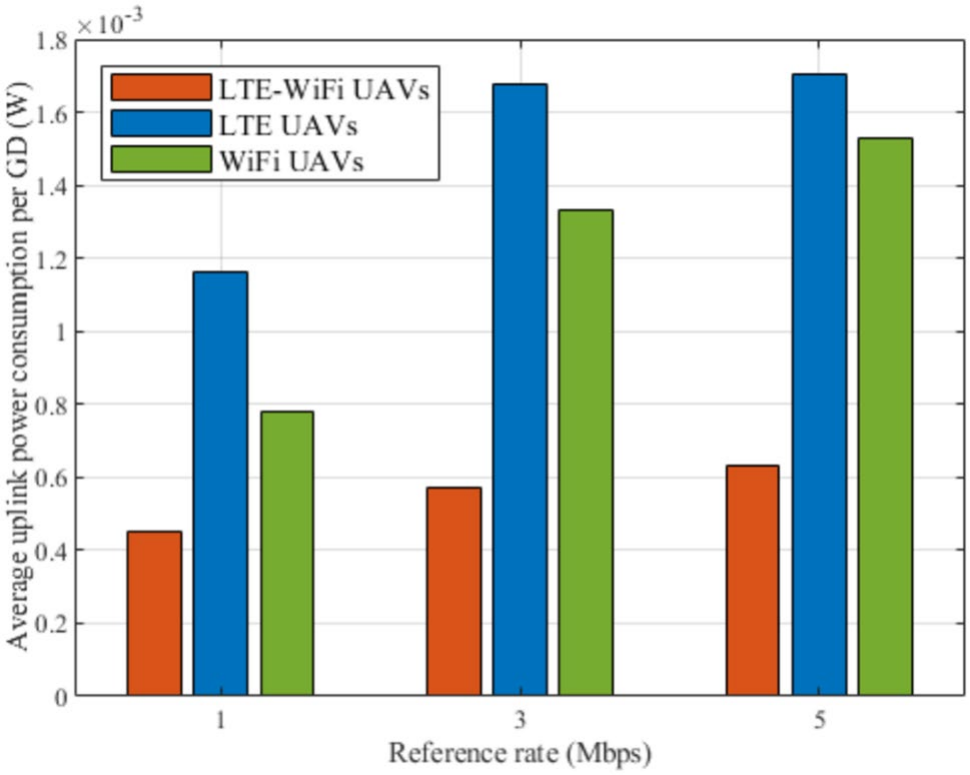
Average GDs uplink power consumption at different reference rates.

**Fig. 17:**
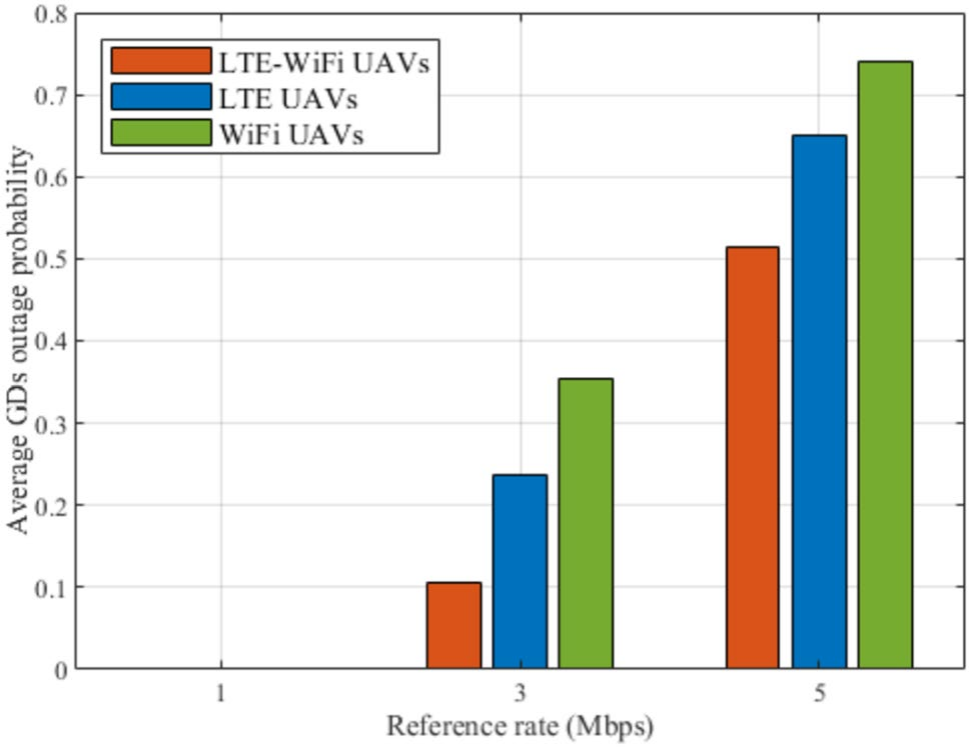
Average GDs outage probability at different reference rates.

**Fig. 18:**
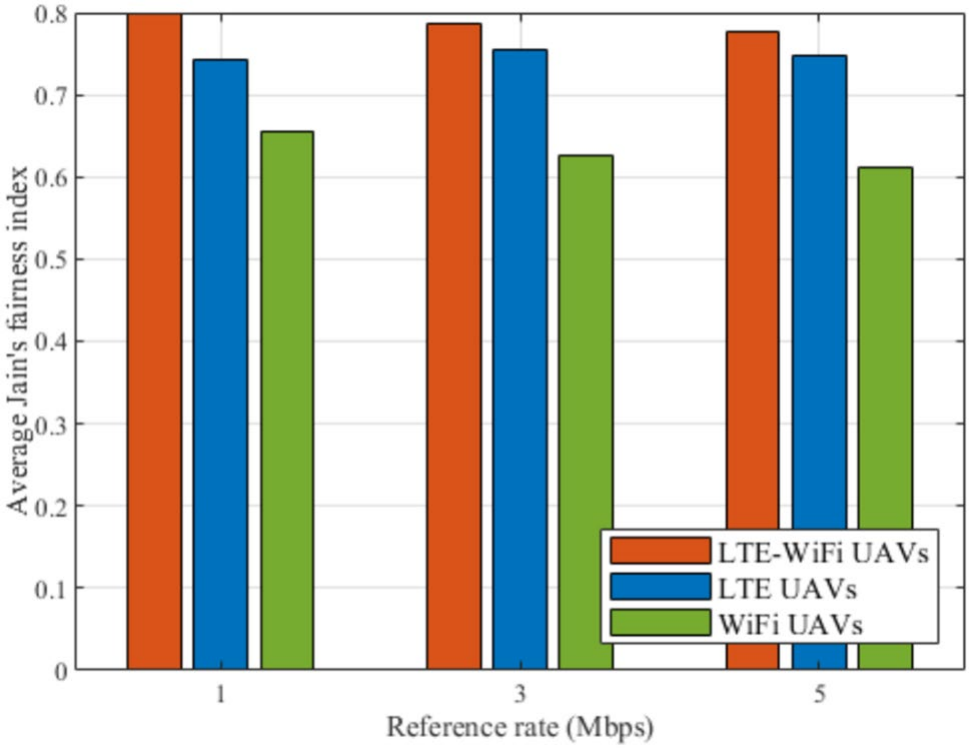
Average Jain’s fairness index at different reference rates.

**Fig. 19:**
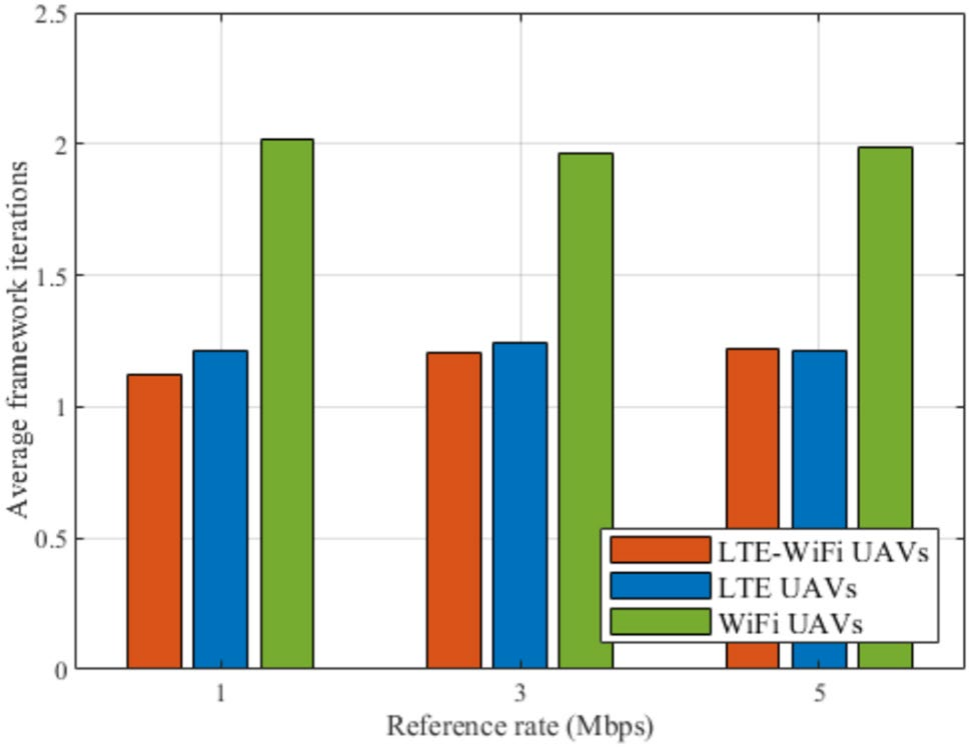
Average framework iterations at different reference rates.

Figures 14, 15, 16, 17, 18, and 19, compare the performance of the different deployments at different reference rate requirements. These results are the average of the values at different numbers of GDs (i.e., from 40 to 100). It can be noticed that the LTE-Wi-Fi UAVs exceeds LTE UAVs and Wi-Fi UAVs at all the different reference rate requirements, considering satisfaction index, downlink data rate, uplink power consumption, outage probability, fairness index, and framework iterations. Generally, increasing the reference rate implies more stringent requirements. Thus, for all the deployments, this decreases the satisfaction index, increases the downlink data rate, uplink power consumption, outage probability, framework iterations, and decreases the Jain’s fairness index.

The increase in the downlink data rate and the uplink power consumption is justified by the effect of increasing the reference rate on the rate term in the satisfaction index. The rate term is a concave function with respect to the reference rate, where a GD gains more benefit of increasing its data rate at relatively low data rate levels significantly, then by increasing the achieved data rate, the benefit gradient gradually decreases until there are no significant benefit gains. Therefore, by increasing the reference rate, all the deployments get more satisfaction gains by achieving higher data rates, in the expense of increasing the uplink power consumption.

Moreover, it can be noticed that LTE UAVs achieves lower performance compared to Wi-Fi UAVs in terms of satisfaction index at 1 Mbps reference rate. This because of the aforementioned characteristics of LTE UAVs, where they tend to achieve higher data rates in the expense of power consumptions, due to their ability to reduce interference by optimizing their locations. And since their achieved data rates are much higher than the reference rate, they gain less satisfaction by increasing their data rates, rather than decreasing their uplink power consumption.

**Table 4:** Performance improvement percentages.

Performance Metric	Scenario	Performance Improvement Percentage					
		Satisfaction index (%)	Downlink rate (%)	Uplink power (%)	Outage probability (%)	Jain’s fairness index (%)	Framework iterations (%)
${R}^{{ref}}=\mathbf{1}\ \text{Mbps}$	LTE UAVs	1	21	61	N/A	8	8
${R}^{{ref}}=\mathbf{3}\ \text{Mbps}$	LTE UAVs	5	15	67	56	5	3
${R}^{{ref}}=\mathbf{5}\ \text{Mbps}$	LTE UAVs	7	16	63	21	4	0
${R}^{{ref}}=\mathbf{1}\ \text{Mbps}$	Wi-Fi UAVs	1	25	42	N/A	22	45
${R}^{{ref}}=\mathbf{3}\ \text{Mbps}$	Wi-Fi UAVs	7	19	58	71	26	39
${R}^{{ref}}=\mathbf{5}\ \text{Mbps}$	Wi-Fi UAVs	13	19	59	31	28	39

It is worth mentioning that, the satisfaction index outperformance percentage does not necessarily imply the same percentage of outperformance in data rate or power consumption, which is illustrated in Table 4. This is due to the non-linear relation between the satisfaction index and the mixture of data rate and power consumption. Since the objective is to maximize the satisfaction index, there might be cases where data rate is chosen to be maximized over power consumption, due to its contribution to the satisfaction index, and vice versa.

For example, in Table 4, the LTE-Wi-Fi UAVs outperformance reaches 13% compared to the Wi-Fi UAVs at a 5 Mbps reference rate in terms of satisfaction index. However, it has the same reflection on the downlink data rate (i.e., 19%), and a slightly better reflection on the uplink power consumption (i.e., 59%), compared to the 7% outperformance in terms of satisfaction index at 3 Mbps reference rate (i.e., 19% in the downlink data rate, and 58% in the uplink power consumption). This is due to the higher contribution of the downlink data rate on the satisfaction index at stringent reference rate requirements, compared to more relaxed reference rate requirements conditions.

Furthermore, in Table 4, it can be noticed that by increasing the reference rate, the outperformance percentage of the LTE-Wi-Fi deployment increases. This reflects the effectiveness of coupling LTE and Wi-Fi base stations on each UAV, especially under stringent requirements.

**Fig. 20:**
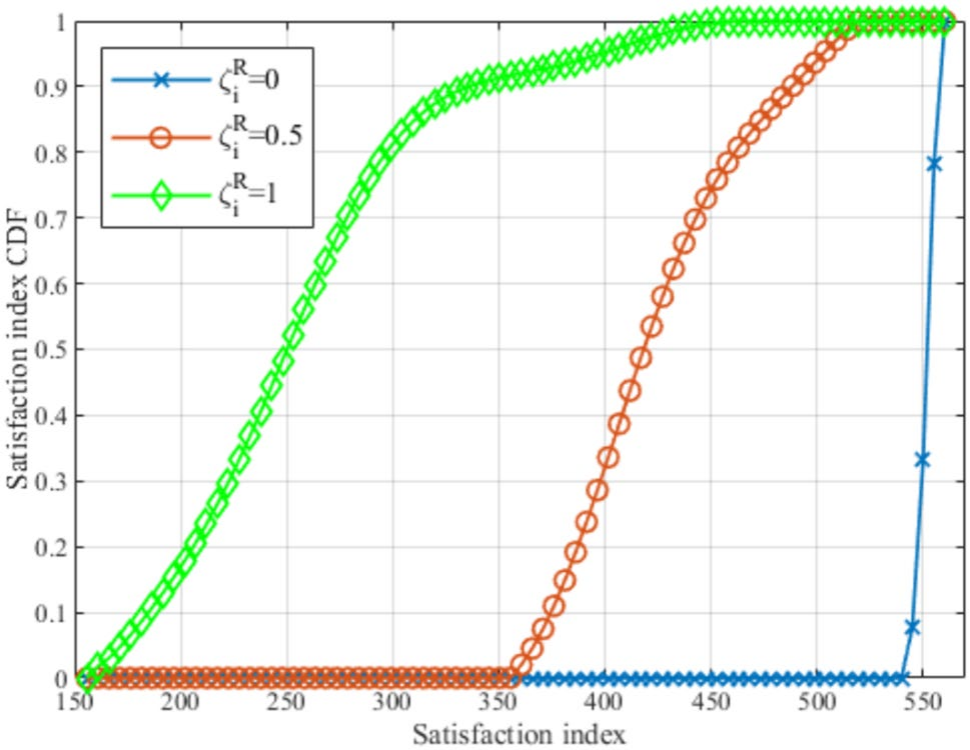
CDF for satisfaction index at different $\zeta _i^R$.

**Fig. 21:**
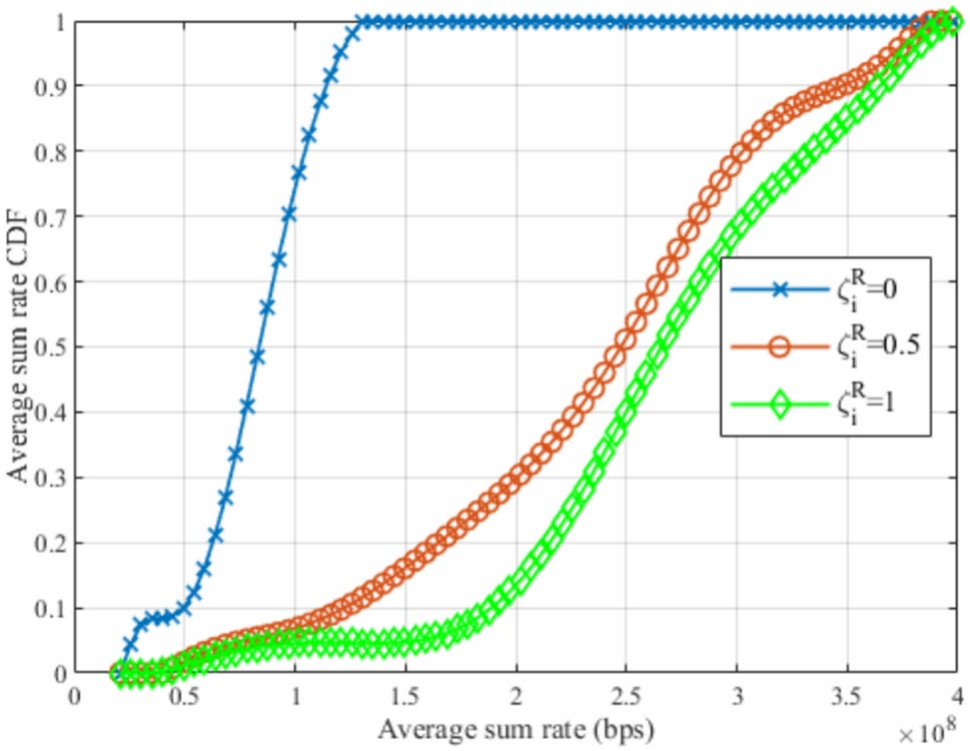
CDF for average sum rate at different $\zeta _i^R$.

**Fig. 22:**
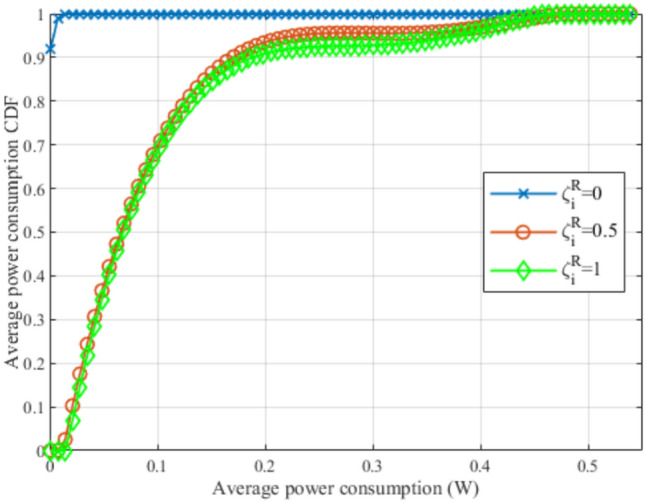
CDF for average power consumption at different $\zeta _i^R$.

**Fig. 23:**
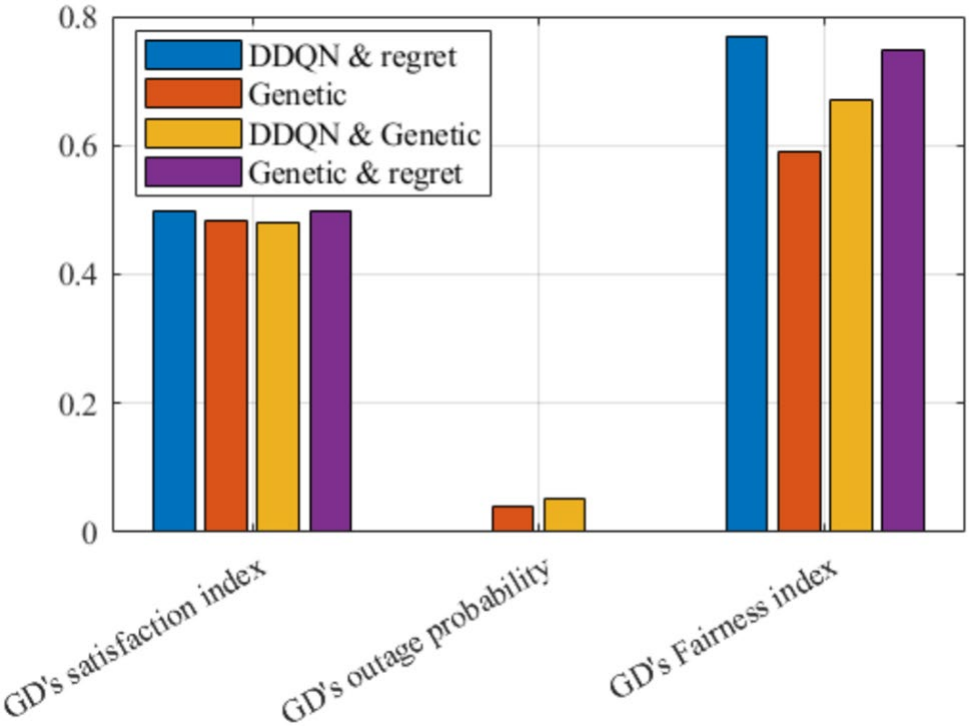
Evaluating the proposed framework compared to genetic algorithm.

Figures 20, 21, and 22, illustrate the effect of changing the weight parameter $\zeta _i^R$ on the average satisfaction index, average downlink sum rate, and average total uplink power consumption, respectively, by measuring the Cumulative Distribution Function (CDF) for each. This reflects the differences between the diverse GDs, where some GDs may be rate oriented (i.e.,$\zeta _i^R=1$), others may be power-oriented (i.e., $\zeta _i^R=0$), and others may be oriented to rate and power at the same time (i.e., $\zeta _i^R=0.5$). It is worth noting that, $\zeta _i^R$ can be any value between 0 and 1, for each GD, which fulfills diverse GDs’ requirements at different times. The values reported in these figures are obtained with $N=80$ and $R^{ref}=5$ Mbps. In Fig. 19, it can be observed that power-oriented GDs (i.e., $\zeta _i^R=0$) have a higher likelihood of achieving higher values of satisfaction index compared to other types of GDs. This implies that higher satisfaction can be achieved by minimizing the power consumption only, this is due to the high reference rate $R^{ref}$ required, which decreases the GDs’ satisfaction from achieving high data rates when $\zeta _i^R=0.5$ and $\zeta _i^R=1$.

In Fig. 22, power-oriented GDs (i.e., $\zeta _i^R=0$) have a significantly higher likelihood of achieving lower values of the average total uplink power consumption compared to other types of GDs. This is because, when $\zeta _i^R=0$, the GDs’ objective is converted into minimizing the uplink power consumption. The same can be observed in Fig. 21, where rate-oriented GDs (i.e., $\zeta _i^R=1$) have a higher likelihood of achieving higher downlink sum-rate values compared to other types of GDs. This is because when the weight parameter $\zeta _i^R=1$, the GDs’ objective is converted into maximizing the downlink data rate.

In order to measure the performance of the proposed framework, DRL algorithm, and regret learning algorithm, we compared it with a meta-heuristic algorithm, which is the genetic algorithm. The genetic algorithm is adopted as a baseline to solve the GD’s association and UAVs location jointly, under the name ‘Genetic’. It is also used to solve the GD’s association only by replacing the regret learning algorithm with it in the framework, under the name ‘DDQN & Genetic’. Also, it is used to solve the UAVs location only by replacing the DDQN algorithm with it in the framework, under the name ‘Genetic & regret’.

Figure 23 compares between the proposed framework ‘DDQN & regret’, ‘Genetic’, ‘DDQN & Genetic’ and ‘Genetic & regret’ in terms of satisfaction index, outage probability, and fairness index. In this comparison we considered the number of GDs to be 40, $\zeta _i^R=0.5$ and $R^{ref}=1Mbps$. Also, the maximum number of stall generation in genetic algorithms is 800. It can be observed that the proposed framework and the ‘Genetic & regret’ achieves better performance compared to others in terms of satisfaction index, outage probability, and fairness index. All the approaches achieve almost the same satisfaction index, since the objective is to maximize the satisfaction index of the GDs. However, the proposed framework performs better than other approaches in the fairness index metric. Not to mention the higher complexity of the genetic algorithm.

The limitations of the proposed work could be the limited capacity of the UAVs’ battery, where each UAV has a limited energy budget. We assume that there are spare and fully charged UAVs that are ready to replace the working UAVs when their energy reaches a predefined critical level, which, accordingly, will increase the operational cost. In addition, the UAVs payload could be considered as another limitation, which will be affected by increasing the number of deployed base stations on it (i.e., LTE base station and Wi-Fi access point). We assume that the UAVs can efficiently carry the Multi-RAT base stations. Moreover, a robust and reliable common control channel for UAVs and control station communication is assumed, which is challenging. Furthermore, a secure UAVs’ data and control communication is assumed, where it is limited by their hardware low computational cost, restricting complex encryption and authentication. All the aforementioned limitations are open challenges that can be studied and investigated in future works.

## Methods

The performance of the proposed work was evaluated by conducting extensive simulations. Matlab R2022a was used to perform these simulations. The GDs locations are randomly distributed using a uniform distribution for each point in each simulation run. Adam Optimizer is used for training network parameters with $2\times 10^{-8}$ learning rate, 64 mini batch size, and 0.0001 $L_2$ regularization factor. The DDQN target network update frequency is set to 10, and the memory replay is 10,0000. Each episode ends when the target with the highest reward is successfully founded or the total number of episodes is reached. The regret learning algorithm iterations end after reaching the maximum number of iterations. The proposed framework is terminated whenever there is no increase in the SER. The UAVs are assumed to have an infinite energy (i.e., stand-by UAVs, or energy harvesting are assumed to be used when UAVs run out of battery).

## Data Availability

The source files/datasets used and/or analyzed during the current study available from the corresponding author on reasonable request.
